# Responses of carbon dynamics to grazing exclusion in natural alpine grassland ecosystems on the QingZang Plateau

**DOI:** 10.3389/fpls.2022.1042953

**Published:** 2022-11-11

**Authors:** Tianyuan Liu, Jian Sun, Youchao Chen, Tiancai Zhou, Hua Shang, Yingxin Wang, Junxi Wu, Qi Mi, Jintao Zhang, Wen He, Le Sun

**Affiliations:** ^1^ State Key Laboratory of Earth System Resources and Environment of Tibetan Plateau, Institute of Tibetan Plateau Research, Chinese Academy of Sciences, Beijing, China; ^2^ College of Environmental and Resource Sciences, Zhejiang A&F University, Hangzhou, China; ^3^ Department of Ecology, Evolution and Natural Resources, Rutgers, The State University of New Jersey, New Brunswick, NJ, United States; ^4^ Research Institute of Forestry, Chinese Academy of Forestry, Beijing, China; ^5^ Key Laboratory of Ecosystem Network Observation and Modelling, Institute of Geographic Sciences and Natural Resources Research, Chinese Academy of Sciences, Beijing, China

**Keywords:** QingZang Plateau, alpine grasslands, grazing exclusion, carbon dynamics, meta-analysis

## Abstract

In the context of “Carbon Emissions Peak” and “Carbon Neutrality”, grazing exclusion (GE) has been applied widely to rehabilitate degraded grasslands and increase carbon sequestration. However, on the QingZang Plateau (QZP), the impacts of GE on the carbon dynamics of alpine grasslands are poorly understood, particularly at a regional scale. To fill this knowledge gap, we evaluated the responses of carbon sequestration to GE in different alpine grasslands across QZP by using meta-analysis. The effects of GE on ecosystem carbon fractions were dependent on GE duration, grassland types and climate factors. Specifically, our results indicated that GE had more obviously positive effects on carbon stock across the alpine meadow than the alpine steppe. However, when considering different GE duration, the longer duration of GE was more effective for increasing ecosystem carbon sequestration (*R* ^2^ = 0.52, *P*<0.0001) in the alpine steppe. Our results further demonstrated that annual mean precipitation (AMP) and temperature (AMT) began to dominate ecosystem carbon sequestration after three years of GE duration across the alpine meadow; and AMP was an important climate factor limiting ecosystem carbon sequestration (*R* ^2^ = 0.34, *P*<0.01) in the alpine steppe. In terms of plant carbon fraction, GE generated continuous positive effect (*P*<0.05) on aboveground biomass with the increased GE duration in the alpine meadow, while this positive effect disappeared after the 8th year of GE duration. And no positive effects were found on belowground biomass in the 11th year in alpine steppe. For soil organic carbon (SOC), there existed periodic fluctuations (increased and then decreased) on SOC in response to GE. For microbial biomass carbon, there were no obvious trends in response to GE duration. In general, we highlighted that the responses of different carbon fractions (plant-soil-microbe) to GE were non-uniform at spatial and temporal scales, thereby we should adopt different carbon management practices for sustainable development of different grasslands.

## Introduction

Global warming induced by the increasing emission of carbon dioxide is receiving great attention all over the world (Mullan et al., 2019; Xue et al., 2021; Zhou et al., 2021). At the General Assembly of the United Nations in September 2020, China made it clear that it would reach the “Carbon Emissions Peak” by 2030 and strive to achieve “Carbon Neutrality” by 2060 (Yuan et al., 2022; Zhao et al., 2022). China has a large area of degraded grassland (Li et al., 2008) with huge potential for increasing carbon sequestration. It is reported that the grasslands on the QingZang Plateau (QZP) have degraded severely (Sun et al., 2021; Sun et al., 2022). Overgrazing is considered as one of the primary causes leading to large-scale grassland degradation across QZP, while ecological restoration projects might have great potential effects to increase carbon sinks (Sun et al., 2019). Grazing, trampling and excessive digging by livestock could damage the aboveground parts of vegetation, which generate negative effects on the nutrients cycles of alpine ecosystem (Sun et al., 2018; Yuan et al., 2020), consequently aggravate the grassland degradation (He et al., 2008). To restore the degraded alpine grassland, grazing exclusion (GE) with fences has been widely adopted by the local government since 2003 (Sun et al., 2020a).

Although GE, as a kind of ecological restoration project on the QZP, might have great potential to increase carbon sequestration, the influences are far from conclusive. Specifically, in terms of plant biomass, some studies observed that GE increased the aboveground biomass (AGB) (Liu and Nie, 2012; Yang et al., 2017) and the belowground biomass (BGB) (Du and Gao, 2021; Wang F, et al., 2020), while other studies found that GE could have a relatively neutral effect on AGB (Yuan et al., 2020) or negative impacts on AGB (Yan et al., 2014) and BGB (Wu et al., 2021). A previous study observed that short-term GE increased AGB but decreased BGB in the alpine meadow (Zhu et al., 2021). Other studies found that AGB increased and then decreased (Chen et al., 2019), BGB decreased and then increased (Hong et al., 2015) with the extension of GE duration across the alpine steppe. As for the soil organic carbon (SOC), the GE effects were also inconsistent, including both positive (Wu et al., 2010; Fan et al., 2013) and negative effects (Shi et al., 2013; Wu et al., 2021). Besides, some studies demonstrated that there is a hump-shaped pattern of AGB and SOC in response to GE duration (Li et al., 2018; Cao et al., 2019; Chen et al., 2019). Some studies reported that long-term GE improved SOC across alpine meadow and alpine steppe (Wang F, et al., 2020; Wu et al., 2010), while others found SOC decreased with longer GE duration in the alpine meadow (Fan et al., 2012; Shi et al., 2013). Meanwhile, discrepant results were reported on the responses of microbial biomass carbon (MBC) to GE, with increasing (Wu et al., 2010; Wei et al., 2018), decreasing (Li et al., 2013), and no significant changes (Luan et al., 2014). GE improved MBC (Wu et al., 2010; Du et al., 2020), but other studies found GE reduced MBC (Shi et al., 2013; Zhang et al., 2015) after a longer GE duration across the alpine meadow. Therefore, we elicit the hypotheses that the differences in GE duration, ecosystem type, and environmental condition may strongly affect the influences of GE on carbon dynamics (Su and Xu, 2021; Yuan et al., 2020; Zhang et al., 2021).

In generally, systematic and explicit studies are needed to better understand the effectiveness of GE and its suitable duration for carbon sequestration under different habitat conditions. Herein, we conducted a meta-analysis to evaluate the responses of various carbon fractions to GE across different alpine grasslands on the QZP. We aimed to understand: 1) the responses of carbon dynamics in plants, soil and microbial biomass to GE in alpine grasslands; 2) and revealed the possible causes. We hope our study could provide more information and suggestions for policymakers on how to develop useful management in the future to improve the grassland carbon sink in the global alpine area.

## Materials and methods

### Study area

The QZP (73°19′-104°47′E, 26°00′-39°47′N) (**Supplementary Figure S1**) is the highest plateau worldwide with an average altitude over 4000 m (Qin et al., 2018), which includes Qinghai Province, Tibet Autonomous Region, and part of Sichuan Province, Gansu Province, Xinjiang Autonomous Region and Yunnan Province (Fu et al., 2021). The QZP is characterized with a fragile environment, and is extremely sensitive to global change (Yang et al., 2022). Alpine meadow and alpine steppe are the main grassland ecosystems across QZP (Duan et al., 2021). The main species are *Stipa purpurea* and *Festuca ovina* in the alpine steppe, and *Kobresia pygmaea* and *Kobresia tibetica* in the alpine meadow (Wang et al., 2022).

### Data collection

ISI Web of Science (https://www.webofscience.com/) and China National Knowledge Infrastructure (CNKI) (https://www.cnki.net/) were used to collect papers published from 1991 to 2021 on GE experiments across the QZP. We used the keywords of “Qinghai Plateau, Tibetan Plateau and QingZang Plateau” in combination with “grazing exclusion” or “fence”, respectively, to screen the articles that meet our criteria, and then 315 articles (**Supplementary Table S1**) were finally used in our study. Paper selections were based on the following criteria: (1) the experimental data must have been collected from field experiments (grazing vs. GE) on the QZP; (2) the duration of GE lasted at least one year; (3) there were no other treatments (e.g., warming, burning or mowing) in the field studies. Data, including AGB, BGB, total biomass (TB), SOC, MBC, and site information (longitude, latitude, annual mean precipitation (AMP), temperature (AMT), and ecosystem types) were collected from each study. In addition, the SOC data were grouped according to the soil depth of 0–10 cm, 10–20 cm and > 20 cm, respectively. The data we collected were either directly from the tables or *via* using the GetData Graph Digitizer software (ver.2.20, www.getadata-graph-digitizer.com/) to extract the data points from the documentary figures.

### Data analysis

Meta-analysis (Hedges et al., 1999) was carried out *via* MetaWin 2.1 to evaluate the response of carbon fractions (AGB, BGB, TB, SOC and MBC) to GE. We computed the natural log converted response ratio (RR) using Eq1:


$$RR=Ln({\bar{X}}_{t}/{\bar{X}}_{c})$$

where ${\bar{X}}_{t}$ and ${\bar{X}}_{c}$ represent the average value of various carbon fractions (AGB, BGB, TB, SOC or MBC) in the GE and grazing group, respectively.

RR’s variance (v) was calculated by Eq2:


(2) $$v=\frac{S_{t}^{2}}{n_{t}{\bar{X}}_{t}^{2}}+\frac{S_{c}^{2}}{n_{c}{\bar{X}}_{c}^{2}}$$

where n_t_ and n_c_ denote the sample sizes of GE and grazing group. S_t_ and S_c_ denote the standard deviations of the concerned carbon fractions (AGB, BGB, TB, SOC and MBC) of GE and grazing group, respectively.

The mean response ratio (RR_++_) was calculated by following Eq3:


(3) $$RR_{++}=\frac{\sum_{i=1}^{m}\sum_{j=1}^{k}w_{ij}RR_{ij}}{\sum_{i=1}^{m}\sum_{j=1}^{k}w_{ij}}$$

where the reciprocal of the variance (w=1/v) is the weight (W) of each RR (Zhan et al., 2019). A random effect model was used to calculate the RR++ and its 95% bootstrap confidence intervals (CIs). If CIs were not through zero, the statistical result was considered to be significant; otherwise, it was insignificant.

We used the composite index of carbon stock function (CSFI) to indicate the composite carbon stock (AGB, BGB and SOC) of the ecosystem across the alpine meadow and the alpine steppe (Zhang et al., 2021), ΔCSFI and ΔZ(i) are the relative increment of composite and single carbon stock function response to GE, Eq4:


(4) $$\{\begin{array}{c} CSFI=\sum_{i=1}^{3}Z\left(i\right)/3\\ Z\left(i\right)=\left(x_{i}-x_{mean}\right)/std\\ \Delta CSFI=CSFI_{GE}-CSFI_{G}\\ \Delta Z\left(i\right)=Z{\left(i\right)}_{GE}-Z{\left(i\right)}_{G} \end{array}$$

where x_i_ is the observation value of the single carbon stock function i (i is AGB, or BGB, or SOC), and Z(i) represents the standardization of x_i_. x_i_, x_mean_, and std represent the measured value, or the mean value, or the standard deviation of carbon stock function i, respectively. CSFI_GE_ and Z(i)_GE_ represent carbon stock functions in the GE group, while CSFI_G_ and Z(i)_G_ represent carbon stock functions in the grazing group.

SigmaPlot for Windows version 10.0 (Systat Software, Inc., Chicago, IL, USA) was used to explore the relationships among the RRs of carbon fractions (AGB, BGB, TB, SOC, and MBC), and also the connections between ΔCSFI and the GE duration, ΔZ(AGB), ΔZ(BGB) as well as ΔZ(SOC) across the alpine meadow and the alpine steppe. The segment function is calculated as follows (Eqs 5-10):


(5) t1=min(t)

(6) t3=max(t)

(7) $$f1\left(t\right)=\frac{y1*\left(T1-t\right)+y2*\left(t-t1\right)}{T1-t1}$$

(8) $$f2\left(t\right)=\frac{y2*\left(T2-t\right)+y3*\left(t-T1\right)}{T2-T1}$$

(9) $$f3\left(t\right)=\frac{y3*\left(t3-t\right)+y4*\left(t-T2\right)}{t3-T2}$$

(10) f=if(t≤T1, f1(t),if(t≤T2,f2(t),regionf(t)))

## Results

### Response patterns of carbon fractions to GE

**Figure 1:**
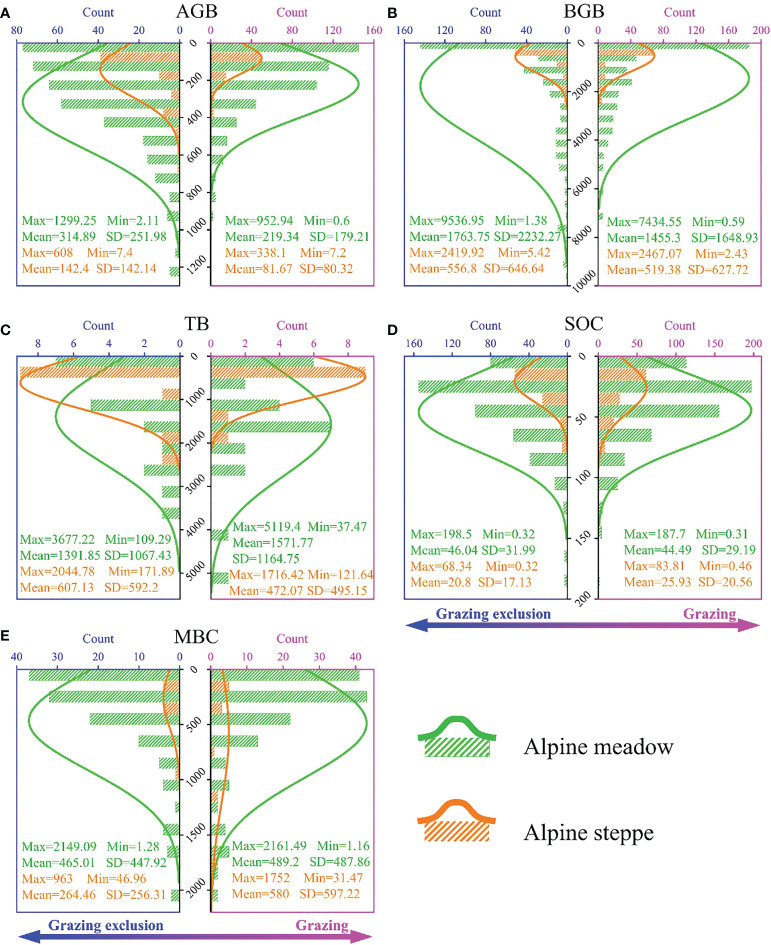
Count distributions of **(A)** aboveground biomass (AGB), **(B)** belowground biomass (BGB), **(C)** total biomass (TB), **(D)** soil organic carbon (SOC) and **(E)** microbial biomass carbon (MBC) in the “grazing” and “grazing exclusion (GE)” group across alpine steppe and meadow. “Max”, “Min”, “Mean” and “SD” represents the maximum value, the minimum value, the average value and the standard deviation, respectively.

In the alpine meadow, the mean value of AGB, BGB, TB, SOC and MBC was 219.34 and 314.89 g/m^2^ (**Figure 1A**), 1455.3 and 1763.75 g/m^2^ (**Figure 1B**), 1571.77 and 1391.85 g/m^2^ (**Figure 1C**), 44.49 and 46.04 g/kg (**Figure 1D**), and 489.2 and 465.01 mg/kg (**Figure 1E**) in the grazing and GE group, respectively. In the alpine steppe, AGB, BGB, TB, SOC and MBC with the mean value was 81.67 and 142.4 g/m^2^ (**Figure 1A**), 519.38 and 556.8 g/m^2^ (**Figure 1B**), 472.07 and 607.13 g/m^2^ (**Figure 1C**), 25.93 and 20.8 g/kg (**Figure 1D**), and 580 and 264.46 mg/kg (**Figure 1E**) in the grazing and GE group, respectively.

**Figure 2:**
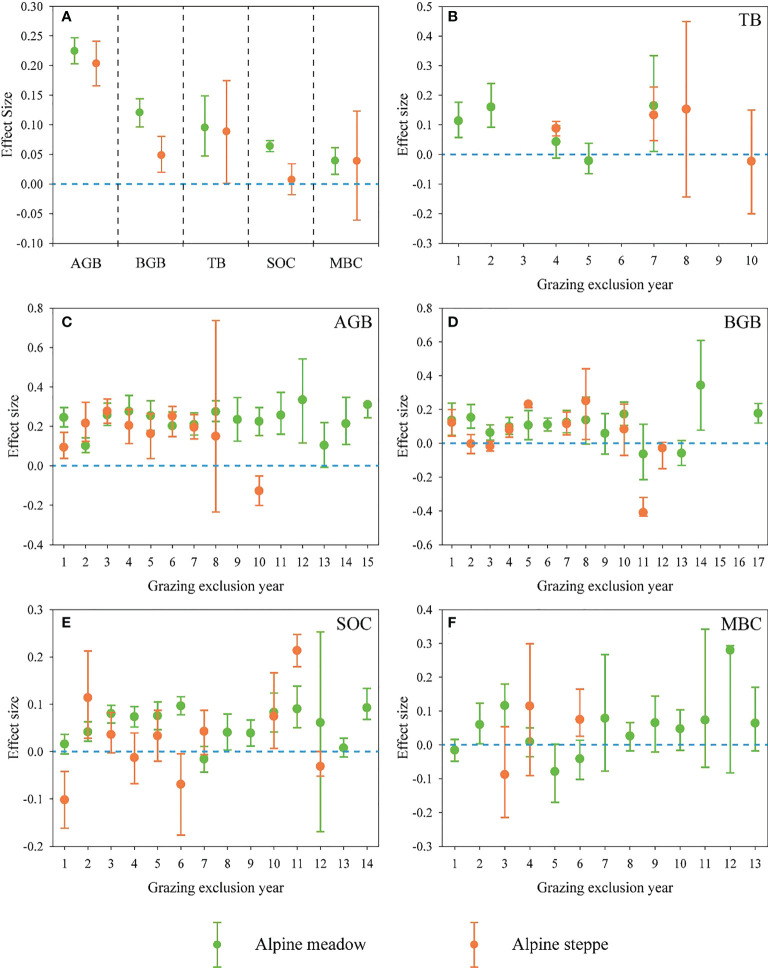
**(A)** Response ratios (RR) of aboveground biomass (AGB), belowground biomass (BGB), total biomass (TB), soil organic carbon (SOC) and microbial biomass carbon (MBC) in response to grazing exclusion (GE) across alpine meadow and steppe. The effects of GE duration on **(B)** TB, **(C)** AGB, **(D)** BGB, **(E)** SOC and **(F)** MBC in alpine meadow and steppe. Error bars indicate 95% confidence intervals (CIs). The effect was not significant if the 95% CIs of the effect size did not overlap with zero.

AGB, BGB, and TB increased significantly with GE in both alpine meadow and alpine steppe (**Figure 2A**). Also, GE had remarkably positive effects on SOC and MBC in the alpine meadow, while it had no significant impacts on SOC and MBC in the alpine steppe (**Figure 2A**). Furthermore, the effects on these carbon fractions varied with duration of GE (**Figure 2B–F**). In both alpine meadow and alpine steppe, there were no obvious trends in TB and MBC with the increased duration of GE (**Figures 2B, F**). In the alpine meadow, GE showed an apparently positive effect on AGB regardless of the duration of GE (**Figure 2C**), however the increase in AGB disappeared at the 8th year of GE across the alpine steppe (**Figure 2C**). Besides, no obvious effects were found on BGB at the 11th year of GE in all of these grasslands (**Figure 2D**).

**Figure 3:**
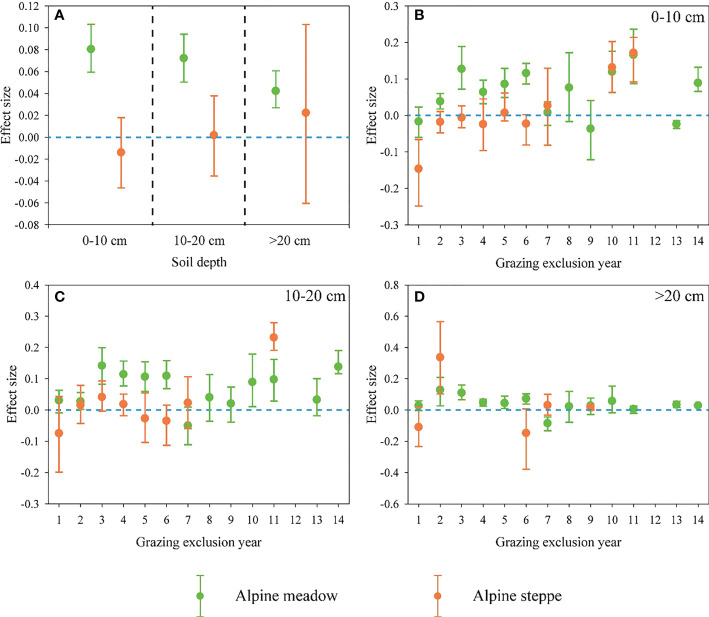
**(A)** Response ratios (RR) of soil organic carbon (SOC) for three soil depths (0–10, 10–20 and > 20 cm) in response to grazing exclusion (GE) across alpine meadow and steppe. The effects of GE duration on SOC at the soil depth of **(B)** 0–10, **(C)** 10–20 and **(D)** > 20 cm in alpine meadow and steppe. Error bars indicate 95% confidence intervals (CIs). The effect was not significant if the 95% CIs of the effect size did not overlap with zero.

GE had significant impacts on SOC across the three soil depths in the alpine meadow, and the response ratio of SOC decreased with increasing soil depths (**Figure 3A**). Nevertheless, there were no significant effects of GE on SOC in the alpine steppe regardless of the soil depths (**Figure 3A**). In addition, in different soil depths, we found that there existed periodic fluctuations (increased and then decreased) on SOC in response to GE (**Figures 3B–D**).

### The response ratios of carbon sequestration to GE

**Figure 4:**
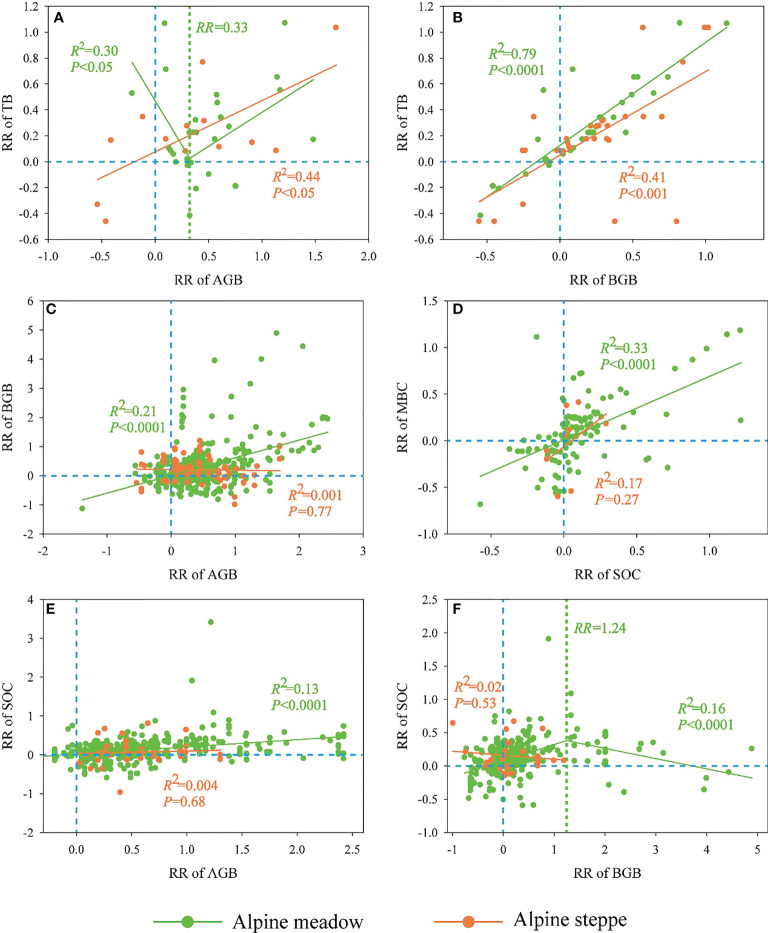
Relationships of response ratios (RR) between aboveground biomass (AGB), belowground biomass (BGB), total biomass (TB), soil organic carbon (SOC) and microbial biomass carbon (MBC) in response to grazing exclusion (GE) across alpine meadow and steppe.

In the alpine meadow, significantly positive relationships between RR of TB and RR of BGB (R^2^ = 0.79, P<0.0001, **Figure 4B**), RR of AGB and RR of BGB (R^2^ = 0.21, P<0.0001, **Figure 4C**), MBC’s RR and SOC’s RR (R^2^ = 0.33, P<0.0001, **Figure 4D**), and AGB’s RR and SOC’s RR (R^2^ = 0.13, P<0.0001, **Figure 4E**) were observed. Besides, the RR of TB firstly decreased and then increased with the increase in the RR of AGB (R^2^ = 0.30, P<0.05, **Figure 4A**), and the turning point of RR was 0.33. On the contrary, the RR of SOC firstly increased and then decreased with the increased RR of BGB (R^2^ = 0.16, P<0.0001, **Figure 4F**), and the RR inflection point was 1.24.

In the alpine steppe, the RR of TB was positively correlated with the RR of both AGB (R^2^ = 0.44, *P*<0.05, **Figure 4A**) and BGB (R^2^ = 0.41, *P*<0.001, **Figure 4B**). Nevertheless, all of the relationships between AGB’s RR and BGB’s RR (**Figure 4C**), MBC’s RR and SOC’s RR (**Figure 4D**), and SOC’s RR and AGB’s RR (**Figure 4E**) were all not significant.

### The relationships of ΔCSFI with carbon fractions, ecosystem types, climatic factors and GE duration

**Figure 5:**
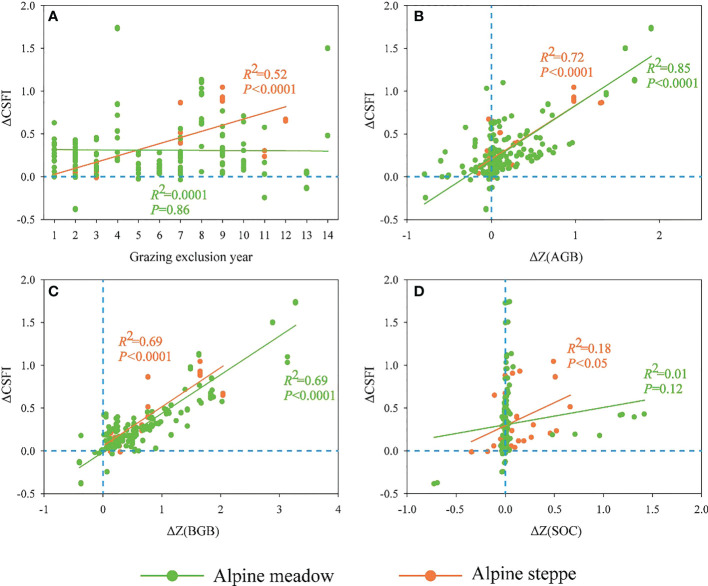
The relationships between ΔCSFI and **(A)** the duration of grazing exclusion (GE), **(B)** ΔZ(AGB), **(C)** ΔZ(BGB), and **(D)** ΔZ(SOC). The CSFI, AGB, BGB and SOC represent the composite index of carbon stock function, aboveground biomass, belowground biomass and soil organic carbon, respectively.

**Figure 6:**
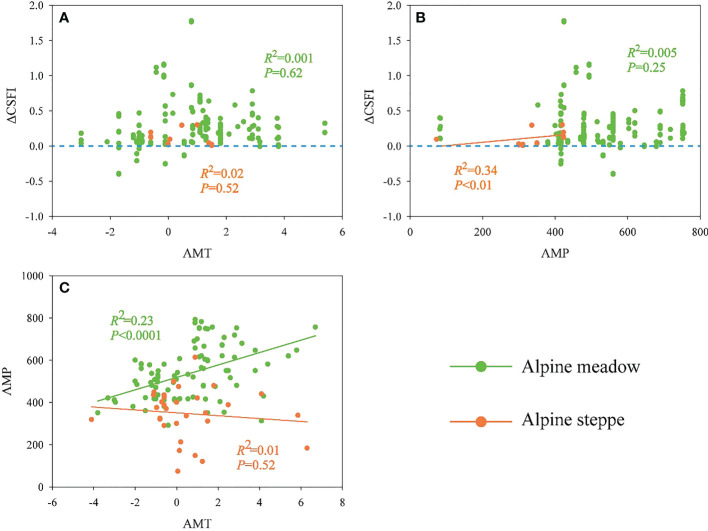
The relationships between **(A)** ΔCSFI and annual mean temperature (AMT), **(B)** ΔCSFI and annual mean precipitation (AMP) and **(C)** annual mean temperature (AMT) and annual mean precipitation (AMP) across alpine meadow and steppe. CSFI represents the composite index of carbon stock function.

There was a significantly increasing tendency of ΔCSFI with the increased GE duration in the alpine steppe (R^2^ = 0.52, *P*<0.0001, **Figure 5A**), while the duration of GE had no significant effect on ΔCSFI in the alpine meadow (R^2^ = 0.0001, *P*=0.86, **Figure 5A**). Both of ΔZ(AGB) and ΔZ(BGB) had significant positive impacts on ΔCSFI across the alpine meadow (R^2^ = 0.85, *P*<0.0001, **Figure 5B**; R^2^ = 0.69, *P*<0.0001, **Figure 5C**) and the alpine steppe (R^2^ = 0.72, *P*<0.0001, **Figure 5B**; R^2^ = 0.69, *P*<0.0001, **Figure 5C**). Also, ΔCSFI was positively correlated to ΔZ(SOC) (R^2 =^ 0.18, *P*<0.05, **Figure 5D**) and AMP (R^2^ = 0.34, *P*<0.01, **Figure 6B**) in the alpine steppe. ΔCSFI was not significantly correlated to AMT in alpine grasslands (**Figure 6A**). In addition, the AMT was significantly and positively correlated with the AMP in the alpine meadow (R^2^ = 0.23, *P*<0.0001, **Figure 6C**). In the alpine meadow, ΔCSFI was significantly correlated with AMP and AMT (*P*<0.05) at the 4^th^ to 6^th^ and 9^th^ to 11^th^ year of GE duration (**Supplementary Table S2**).

## Discussion

### Responses of carbon fractions to GE in alpine grasslands

GE generally improved carbon fractions of plant-soil-microbe across alpine grassland (**Figure 2A**), which is in line with previous studies (Hong et al., 2015; Du et al., 2020; Liu et al., 2020; Li et al., 2021). This is because GE reduces the flow of material and energy from the grassland ecosystem to livestock (Deng et al., 2017). Moreover, GE can prevent grassland from being eaten and trample by livestock, which would facilitate the restoration of plant photosynthesis function, improve grassland productivity as well as soil aeration, increase the accumulation of litter and humus, and further improve soil quality (Hao and He, 2019; Yang et al., 2018; Yu et al., 2021). Specifically, GE showed more positive effects on these carbon fractions in the alpine meadow than in the alpine steppe (**Figure 2A**). Different habitat conditions were the key factors that controlled the influences of GE on carbon storage between alpine meadow and alpine steppe, with more carbon could be accumulated in relatively humid regions (alpine meadow) than that in the relatively arid regions (alpine steppe) (Su and Xu, 2021; Sun et al., 2020b). Both in the alpine meadow and alpine steppe, GE was more effective in promoting AGB than BGB (**Figure 2A**), which is consistent with other studies (Xiong et al., 2014; Hong et al., 2015; Zhu et al., 2021). According to previous studies, plants would allocate more biomass to belowground parts to minimize the loss caused by grazing (Diaz et al., 2007; Yan et al., 2013). Interestingly, GE was less effective in increasing SOC than plant biomass in both alpine meadow and steppe (**Figure 2A**). This is largely attributed to the fact that, unlike plant biomass, soil properties are relatively stable since they are mainly derived from the decomposition of litter, and the decomposition process by soil microbes is relatively slow in the dry and cold QZP area (Chen et al., 2019; Zhan et al., 2019). In addition, the positive effect of SOC had no obvious synergistic raised trend with the increasing positive effect of plant biomass during GE (**Figures 4E, F**).

Continuous beneficial effects for community biomass of the alpine meadow and the alpine steppe disappeared with longer GE duration (**Figures 1C**, **2C, D**), consistent with previous studies (Li et al., 2018; Chen et al., 2019). Explanations for the results are as follows: Firstly, the grassland community tends to be stable and the plant diversity may decrease with the increasing duration of GE (Tenzin et al., 2018; Chen et al., 2019); Secondly, the formation of the biological soil crusts on the surface of grasslands caused by the removal of livestock disturbance might limit the input of rainfall and litter, which inhibited plant growth (Zhao et al., 2006; Zhang et al., 2008). Thirdly, the increased GE duration might thicken the litter layer, and further suppress plant photosynthesis (Su and Xu, 2021); Lastly, the species composition might change after long-term grassland succession leading to the degradation of plant community structure (Zhang et al., 2021).

Besides, the positive response of SOC to GE was highest in the soil depth of 0-10 cm (**Figure 3A**). The main reason might be that GE directly regulates the plant and litter biomass, and the contribution of litter and roots to soil nutrients is mainly concentrated in the surface soil (Wang et al., 2012). Furthermore, the unique, cold and harsh alpine climate of QZP results in a relatively slow decomposition rate which could increase the accumulation of SOC in the surface soil (Wang et al., 2002; Yang et al., 2008). Notably, there were periodic fluctuations of SOC in response to increased GE duration (**Figures 2E**; **3B–D**), since self-regulation and relatively stable soil status might be necessary for maintaining the stability of alpine ecosystems (Li et al., 2003).

### Inherent mechanisms of carbon fractions in response to GE in alpine grasslands

The relationship between the RR of AGB and the RR of BGB was significantly positive (*P*<0.0001) in the alpine meadow, but there was no significant relationship in the alpine steppe (**Figure 4C**). The reason might be that plants tend to maintain a relatively stable pattern between the aboveground and the belowground parts in the alpine meadow (Enquist and Niklas, 2002). However, the RR of BGB was relatively stable in the alpine steppe, and the stability of BGB might arise from the specific survival strategies of the alpine steppe community. Compared with the alpine meadow, the alpine steppe in a relatively arid and barren soil environment was expected to struggle for its survival (Shipley and Meziane, 2002; Zeng et al., 2014; Zhou et al., 2020). In other words, the alpine steppe in a hostile environment was unable to balance the aboveground and the belowground parts. Moreover, the RR of SOC increased with increasing RR of BGB within the threshold level of 1.24 (**Figure 4F**), since BGB was the vital source of SOC (Li et al., 2014; Su and Xu, 2021). It is worth noting that the RR of SOC is negatively related to the RR of BGB beyond this threshold (**Figure 4F**), the increased root biomass resulted in more root exudates secreting into the soil, and further promoted the microbial activities and the turnover of SOC (Zhan et al., 2020). In addition, the RR of MBC was significantly correlated with the RR of SOC in the alpine meadow, while no correlation was found across the alpine steppe (**Figure 4D**). Previous studies had confirmed that the turnover of soil carbon might be constrained under harsh habitat conditions with limited soil resources in the alpine steppe (Zhan et al., 2019), although the microbial growth and reproduction directly depend on the soil resources (Sun et al., 2017).

With the increasing duration, GE had an obvious positive effect on ΔCSFI in the alpine steppe, while had no significant effect on ΔCSFI in the alpine meadow (**Figure 5A**). The findings indicated that it could be more effective for ecosystem carbon sequestration in alpine steppe after longer duration of GE. Compared with alpine meadow, alpine steppe with harsher habitat conditions might need a longer recovery time (Sun et al., 2020a). Furthermore, it might be ascribed to the difference in water and heat availability between the alpine meadow and the steppe, since the AMP varied with the AMT in the alpine meadow, while no significant relationship was found between AMP and AMT in the alpine steppe (**Figure 6C**). It has been proved that more precipitation and higher temperature would facilitate ecosystem productivity and carbon accumulation in the alpine meadow. However, relatively limited precipitation and high temperature in the alpine steppe were expected to generate strong water pressure on plants, which resulted in low carbon accumulation (Bai et al., 2004; Sun et al., 2020a). Additionally, ΔCSFI increased with the increasing precipitation across the alpine steppe (**Figure 6B**), indicating that precipitation is an important climatic factor that limits ecosystem carbon sequestration responding to GE in the alpine steppe. Therefore, in contrast to the alpine steppe, favourable water and heat conditions were beneficial for grassland restoration in the alpine meadow. Moreover, variations of CSFI were more positively correlated with variations of AGB (**Figure 5B**) and BGB (**Figure 5C**) than SOC (**Figure 5D**) across the alpine meadow and the steppe, indicating that the plant biomass, not the soil, acts as the critical contributor of ecosystem carbon stock in response to GE across alpine grasslands. In the alpine region, the relatively low temperature has limited the material cycles of soil and the decomposition of litter, resulting in a slow response rate of SOC to GE (Zhu et al., 2016; Chen et al., 2019). And GE directly promotes grassland productivity by protecting the plant from being eaten by livestock, and the low turnover of soil makes the response of soil to GE lag behind that of the plant.

### Implications for GE management

Although our results indicated that GE is an effective way to increase carbon sequestration, long-term GE might reduce the benefits of grassland restoration and weaken the potential carbon sequestration. Consequently, GE could be removed appropriately at that time. Since the habitat conditions are diverse among different ecosystem types (e.g., alpine meadow and alpine steppe), the potential of carbon sequestration and the corresponding mechanisms might be different in response to GE. Therefore, the management of GE using fences should be treated differently in different habitat conditions. Especially, according to our study, a longer duration of GE is conducive to the increase of ecosystem carbon sequestration in the alpine steppe (**Figure 5A**), and GE is more effective for improving carbon stock in the alpine meadow (**Figure 2A**) but only in the short-term GE (**Figure 5A**). GE could increase plant biomass more obviously than SOC and MBC (**Figure 2A**), and plant carbon fraction might play the dominant role in the ecosystem carbon sequestration during GE (**Figures 5B, C**). Furthermore, climatic factors instead of GE duration might dominate ecosystem carbon sequestration responding to GE when GE reaches a certain duration, for example, precipitation and temperature began to have obvious effects on ΔCSFI after about three years of GE duration across the alpine meadow based on our study (**Supplementary Table S2**). In the future, a sustainable observation network of GE should be constructed across QZP to define an optimal duration of GE scientifically based on a unified standard on the diverse alpine grassland ecosystems across the QZP to make the research conclusion more accurate and reliable.

## Conclusion

In conclusion, we found that the effect of GE on carbon sequestration depends on GE duration, ecosystem type and climate factors. Without considering the duration of GE, GE was more effective in improving plant biomass, soil organic carbon, and microbial biomass carbon across the alpine meadow in general. When considering the GE duration, increasing duration generated a more positive effect on the ecosystem carbon sequestration in the alpine steppe. Moreover, precipitation and temperature began to dominate ecosystem carbon sequestration after three years of GE duration across the alpine meadow. The scientific duration of GE should be adopted in the different grassland ecosystems as well as to reach the goals of “Carbon Emissions Peak” and “Carbon Neutrality”, constructing a sustainable observation network of GE across QZP based on a unified standard in the future is highly recommended.

## Data availability statement

The original contributions presented in the study are included in the article/**Supplementary Material**. Further inquiries can be directed to the corresponding author.

## Author contributions

TL and JS conceived the study. TL, JS, QM, JZ, WH, and LS collected and analysed the data. TL, JS, QM, JZ, WH, and LS drew the graphs. TL and JS wrote the manuscript. TL, JS, YC, TZ, HS, YW, and JW reviewed and revised the manuscript. All authors contributed to the article and approved the submitted version.

## Funding

This research was jointly funded by the National Natural Science Foundation of China (Grant No. 41871040), the Joint Research Project of Three-River- Resource National Park funded by the Chinese Academy of Sciences and Qinghai Provincial People’s Government (LHZX-2020-08), and the Second Scientific Expedition to the Qinghai-Tibet Plateau (Grant No.2019QZKK0405).

## Conflict of interest

The authors declare that the research was conducted in the absence of any commercial or financial relationships that could be construed as a potential conflict of interest.

## Publisher’s note

All claims expressed in this article are solely those of the authors and do not necessarily represent those of their affiliated organizations, or those of the publisher, the editors and the reviewers. Any product that may be evaluated in this article, or claim that may be made by its manufacturer, is not guaranteed or endorsed by the publisher.
