# Supplementary material for: Responses of carbon dynamics to grazing exclusion in natural alpine grassland ecosystems on the QingZang Plateau
Source: Front Plant Sci. 2022 Nov 11;13:1042953. doi: 10.3389/fpls.2022.1042953 (PMC9692098; doi:10.3389/fpls.2022.1042953)
Supplement: Supplementary file 1 [file DataSheet_1.docx]

**Table S1** List of all the following references used in the meta-analysis

Bai, M. Y. Z., Ren, D. Z., Wang, R. H., Pan, G., Zhou, Y. Z. 2017. The effect of enclosure closure on plant diversity in Tibet wetland. Heilongjiang Animal Science and Veterinary Medicine, (9), 147-149+154.

Bai, W. L., Hu, F. C., Li, C. T. 2015. Study on Enclosure Period and Utilization Mode of Tianzhu County Grassland. Journal of Animal Science and Veterinary Medicine, 34(5), 40-43.

Baranova, A., Oldeland, J., Wang, S. L., Schickhoff, U. 2019. Grazing impact on forage quality and macronutrient content of rangelands in Qilian Mountains, NW China. Journal of Mountain Science, 16(1), 43-53.

Cao, J. J. 2010. Effects of Grassland Management Changes on Soil Organic Carbon and Nitrogen Pools in the Qilian Mountains, China. Gansu Agricultural University.

Cao, J. J., Li, G. D., Adamowski, J. F., Holden, N. M., Deo, R. C., Hu, Z. Y., Zhu, G. F., Xu, X. Y., Feng, Q. 2019. Suitable exclosure duration for the restoration of degraded alpine grasslands on the Qinghai-Tibetan Plateau. Land use policy, 86, 261-267.

Cao, X. J., Hasbagan, G., Hu, G. Z., Gao, Z. Q. 2019. Characteristics of Grassland Degradation in the Qinghai Tibetan Plateau, Based on NDVI3g Data. Chinese Journal of Agrometeorology, 40(02), 86-95.

Chai, X. H., Yao, T., Wang, L. D., Sun, G. Z., Pu, X. P. 2014. Impact of enclosure on soil microbial characteristics of alpine grassland. Grassland and Turf, 34(05), 26-31.

Chao, Z. G., Wang, S. P., Xu, G. P., Hu, Y. G., Zhang, Z. H. 2008. Effect of Fencing on Community Structure and Distribution Patterns of Main Populations in Degraded Kobresia humilis Meadow. Acta Botanica Boreali-Occidentalla Sinica, 28(11), 2320-2326.

Chen, D. D., Sun, D. S., Zhang, S. H., Du, G. Z., Shi, X. M., Wang, X. T. 2011. Soil N Mineralization of An Alpine Meadow in Eastern Qinghai-Tibetan Plateau. Acta Agrestia Sinica, 19(3), 420-424.

Chen, D. D., Sun, D. S., Zhang, S. H., Tan, Y. R., Du, G. Z., Shi, X. M. 2011. Effect of grazing intensity on soil microbial characteristics of an alpine meadow on the Tibetan Plateau. Journal of Lanzhou University (Natural Sciences), 47(1), 73-77+81.

Chen, D. M., Zhang, N. N., Liu, L., Zhong, B., Tang, Z. L., Yan, W. C., Xu, L. Y., Sun, G. 2016. The effect of different restoration measures on the desertified alpine grassland in Zoige. Chinese Journal of Applied and Environmental Biology, 22(4), 573-578.

Chen, J. 2015. The responses of ecosystem carbon exchanges to experimental warming and grazing exclusion in a meadow grassland on the northern shore of Qinghai Lake, China. Insitute of Earth Environment,Chinese Academy of Sciences.

Chen, J., Luo, Y. Q., Xia, J. Y., Zhou, X. H., Niu, S. L., Shelton, S., Guo, W., Liu, S. X., Dai, W. T., Gao, J. J. 2018. Divergent responses of ecosystem respiration components to livestock exclusion on the Qinghai Tibetan Plateau. Land Degradation and Development, 29(6), 1726-1737.

Chen, J., Zhou, X. H., Wang, J. F., Hruska, T., Shi.W. Y., Cao, J. J., Zahng, B. C. 2016. Grazing exclusion reduced soil respiration but increased its temperature sensitivity in a meadow grassland on the Tibetan Plateau. Ecology and Evolution, 6(3), 675-687.

Chen, S. F. 2017. Effect of Various Grassland Management on Soil Nutrient of Alpine Grassland in North Tibet. Journal of Shanxi Agricultural Sciences, 45(8), 1315-1317.

Chen, X. P. 2013. Lanzhou University. Effects of Carbon and Nitrogen Additions on the Balances of Carbon and Nitrogen in Alpine Meadow of Northern Tibet. Lanzhou University.

Chen, X. P., Zhang, T., Guo, R. Y., Li, H. Y., Zhang, R., Degen, A. A., Huang, K. W., Wang, X. M., Bai, Y. F., Shang, Z. H. 2021. Fencing enclosure alters nitrogen distribution patterns and tradeoff strategies in an alpine meadow on the qinghai-tibetan plateau. Catena, 197.

Dang, Y. G., Ma, X. Y. 2018. Effects of enclose on the alpine meadow forage yield. Qinghai Prataculture, 27(4), 23-25.

Deng, B. 2012. The study about change of vegetation and C:N:P stoichiometry of soil in different succession stage in alpine grassland. Lanzhou University.

Ding, C. X., Yang, X. X., Dong, Q. M. 2020. Effects of Grazing Patterns on Vegetation,Soil and Microbial Community of Qinghai-Tibetan Plateau. Acta Agrestia Sinica, 28(1), 159-169.

Ding, M. J., Zhang, Y. L., Liu, L. S., Wang, Z. F. 2010. Temporal and Spatial Distribution of Grassland Coverage Change in Tibetan Plateau since 1982. Journal of Nature Resources, 25(12), 2114-2121.

Dong, Q. M., Zhao, X. Q., Ma, Y. S., Shi, J. J., Wang, Y. L., Li, S. X., Yang, S. H., Wang, L. Y., Sheng, L. 2012. Influence of grazing on biomass,growth ratio and compensatory effect of different plant groups in Kobresia parva meadow. Acta Ecologica Sinica, 32(9), 2640-2650.

DorjeDhondub. 2009. A year-round balance providing model of forage for livestock system in the Tibet northern high-cold grassland. Northwest A & F University.

Du, C. J., Gao, Y. H. 2021. Grazing exclusion alters ecological stoichiometry of plant and soil in degraded alpine grassland. Agriculture Ecosystems and Environment, 308.

Du, C. J., Jing, J., Shen, Y., Liu, H. X., Gao, Y. H. 2020. Short-term grazing exclusion improved topsoil conditions and plant characteristics in degraded alpine grasslands. Ecological Indicators, 108, 105680.

Du, K., Kang, Y. K., Zhang, D. G., Su, J. H. 2020. Effects of Different Grazing Patterns on Organic Carbon and Nitrogen Pools in Alpine Meadow in the Qilian Moutains. Acta Agrestia Sinica, 28(05), 1412-1420.

Du, K., Kang, Y. K., Zhang, D. G., Su, J. H. 2021. Effects of different grazing patterns to vegetation characteristics in an alpine meadow on the eastern edge of Qilian Mountains. Grassland and Turf, 41(3), 9-18,25.

Du, W., Sun, N., Zhou, G. Y., Wu, S. M., Nie, C., Li, Y., Liu, Y. H. 2018. Effects of temperature and moisture on nitrogen mineralization under different management practices in alpine meadow. Grassland and Turf, 38(4), 1-11.

Duan, M. J. 2011. Remote Sensing Monitoring of Stipa Purpurea Alpine Grassland Aboveground Biomass under the Grazing Disturbance in Northern Tibet. Chinese Academy of Agricultural Sciences.

Duan, M. J., Gao, Q. Z., Wan, Y. F., Li, Y., Guo, Y. Q., Ganzhu, Z. B., Liu, Y. T., Qin, X. B. 2011. Biomass estimation of alpine grasslands under different grazing intensities using spectral vegetation indices. Canadian Journal of Remote Sensing, 37(4), 413-421.

Duo, J. D. Z., Zhang, Z. Y., Lu, Y., Cao, S. H. 2009. Research on the Improvement of Degraded Natural Grassland in Tibet. Journal of Anhui Agricultural Sciences, 37(12), 5508-5509,5528.

Fan, D. D., Kong, W. D., Wang, F., Yue, L. Y., Li, X. Z. 2020. Fencing decreases microbial diversity but increases abundance in grassland soils on the Tibetan Plateau. Land Degradation and Development, 31(17), 2577-2590.

Fan, Y. J., Hou, X. X., Shi, H. X., Shi, S. L. 2013. Effects of grazing and fencing on carbon and nitrogen reserves in plants and soils of alpine meadow in the three headwater resource regions. Russian Journal of Ecology, 44(01), 80-88.

Fan, Y. J., Hou, X. Y., Shi, H. X., Shi, S. L. 2012. The response of carbon reserves of plants and soils to different grassland managements on alpine meadow of three headwater source regions. Grassland and Turf, 32(05), 41-46,52.

Fu, G., Shen, Z. X. 2017. Grazing alters soil microbial community in alpine grasslands of Northern Tibet. Acta Prataculturae Sinica, 26(10), 170-178.

Fu, G., Shen, Z. X., Zhang, X. Z., Zhou, Y. T., Zhang, Y. J. 2012. Response of microbial biomass to grazing in an alpine meadow along an elevation gradient on the Tibetan Plateau. European Journal of Soil Biology, 52, 27-29.

Fu, G., Zhang, X. Z., Yu, C. Q., Shi, P. L, Zhou, Y. T., Li, Y. L., Yang, P. W., Shen, Z. X. 2014. Response of soil respiration to grazing in an alpine meadow at three elevations in Tibet. The Scientific World Journal, 265142.

Fu, Y., Xiao, J. S., Su, W. J., Xiao, R. X., Zhang, J., Wang, L. J. 2006. The monitor and appraisal of the forage grass growing trend of Qinghai province in 2005. Qinghai Prataculture, 15(1), 2-9.

Gan, Y. M., Luo, Y. J., Zhou, J. F., Zhang, J. H. 2009. Effect of ecological restoration project in northwestern Sichuan on the vegetation community of grassland under desertification. Pratacultural Science, 26(6), 51-56.

Gao, F., Wang, B., Shi, Y. X., Zhang, G. X., Wang, J., Si, G. C., Han, C. H., Yuan, Y. L., Hu, Y. 2017. The response of alpine grasslands ecosystem in the north Tibet to short-term enclosure. Acta Ecologica Sinica, 37(13), 4366-4374.

Gao, Y. C., Chen, H., Luo, P., Wu, N., Wang, G. X. 2007. Effects of grazing intensity on decompositions of two dominant plant species litters in alpine meadow on the Northwester Sichuan. Ecological Science, 26(3), 193-198.

Gao, Y. H. 2007. Study on Carbon and Nitrogen Distribution Pattern and Cycling Process in an Alpine Meadow Ecosystem under Different Grazing Intensity. Chengdu Institute of Biology, Chinese Academy of Sciences.

Gao, Y. H., Zeng, X. Y., Schumann, M., Chen, H., Gao, Y., Zeng, X. 2011. Effectiveness of exclosures on restoration of degraded alpine meadow in the eastern tibetan plateau. Arid Land Research and Management, 25(2), 164-175.

Guo, C. H., Zhang, J., Wang, K. N., Yi, X. D. J., Wu, Y. J., Suo, L. D. 2007. Yearly Dynamics of Biomass and Nutrient Contents in Alpine Grassland. Chinese Journal of Grassland, 29(1), 1-5.

Guo, H. Y., De, K. J., Wang, W., Xu, C. T., Zhang, M., Wei, X. J. 2014. Effect of different fostered measures on primary productivity of Alpine meadow in Sanjiangyuan Region. Chinese Qinghai Journal of Animal and Veterinary Sciences, 44(5), 1-3.

Guo, N., Degen, A. A., Deng, B., Shi, F. Y., Bai, Y. F., Zhang, T., Long, R. J., Shang, Z. H. 2019. Changes in vegetation parameters and soil nutrients along degradation and recovery successions on alpine grasslands of the Tibetan Plateau. Agriculture, Ecosystems & Environment, 284, 106593.

Guo, N., Han, T. H., Wang, J., Han, T., Sun, B. 2010. Ecological Effects of "Restoring Grazing to Grassland Project" in Maqu County. Journal of Desert Research, 30(1), 154-160.

He, G. Y., Sun, H. Z., Shi, X. M., Qi, W., Du, G. Z. 2015. Soil properties of Tibetan Plateau alpine wetland affected by grazing and season. Acta Prataculturae Sinica, 24(4), 12-20.

He, T. 2015. Effect of grazing rates on plant diversity and productivity in northwest Sichuan alpine meadow. Sichuan Agricultural University.

Hong, J. T., Ma, X. X., Wang, X. D. 2016. Leaf meristems: an easily ignored component of the response to human disturbance in alpine grasslands. Ecology and Evolution, 6(8), 2325-2332.

Hong, J. T., Wu, J. B., Wang, X. D. 2015. Effects of grazing and fencing on Stipa purpurea community biomass allocation and carbon,nitrogen and phosphorus pools on the northern Tibet Plateau alpine. Pratacultural Science,32(11), 1878-1886.

Huang, C. H., Peng, F., You, Q. G., Liao, J., Duan, H. C., Wang, T., Xue, X. 2020. The response of plant and soil properties of alpine grassland to long-term exclosure in the northeastern Qinghai–Tibetan Plateau. Frontiers in Environmental Science, 8, 589104.

Huang, G. Z., Xi, Y. L., Zhao, C. Y., Liu, R. X., Yang, J. H., Li, N., Li, W. B. 2020. Effects of enclosure on subalpine grassland community structure and productivity in Qilian Mountains. Journal of Lanzhou University. Natural Science, 56(6), 718-723.

Huang, M. 2021. The Effect of Long-term Grazing Exclusion by Fencing on Vegetation, Soil and Resilience of “Bare Land” Sown Grassland in Three Rivers Source Region on the Tibetan Plateau. Lanzhou University.

Huang, Y., Zhao, C., Yang, Y., Liu, K., Mou, Q. Y., Yang, L., Hu, J. Y. 2021. Coupling Mechanism of Grazing Exclusion Period and Recovery Processes in Alpine Desertification Grassland in Northwest Sichuan. Journal of Sichuan Forestry Science and Technology, 42(1), 60-64.

Jia, Z. F., Ma, X., Xu, C. T., Liu, W. H., Wei, X. X., Lei, S. C. 2019. Effect of short-term enclosure on the vegetation characteristics of a lightly degraded alpine meadow in Guinan County. Prataculturae Science, 36(11), 2766-2774.

Jiang, Y. M., Yao, T., Li, J. H., Chen, L., Liu, H., Liu, T., Tian, Y. L., Zhang, B., Gao, Y. M. 2016. Effects of different management measures on soil microbial biomass in an alpine meadow. Acta Prataculturae Sinica, 25(12), 35-43.

Jiang, Y. M., Yao, T., Tian, Y. L., Yang., H. H., Zhang, J. G., Gao, Y. M., Wei, C. H., Zeng, X. X., Yan, C. X., Zhang, B. 2016. Effect of different management patterns on seasonal dynamics of soil microbial biomass in the alpine meadow. Grassland and Turf, 36(5), 105-110.

Jing, J., Zhang, M. Y., Gao, Y. H. 2021. Effects of enclosure on soil microbial carbon utilization in an alpine steppe. Ecological Science, 40(3), 25–32.

La, C. M., Yang, Z. W., Cheng, W. S. 2012. Affect Study of Fencing on Desertified Grassland Community Features of Qinghai Lake East. Science and Technology of Qinghai Agriculture and Forestry, (1), 23-25,50.

Li, F. X., Li, X. D., Zhou, B. R., Qi, D. L., Wang, L., Fu, H. 2015. Effects of grazing intensity on biomass and soil physical and chemical characteristics in alpine meadow in the source of three rivers. Pratacultural Science, 32(1), 11-18.

Li, H. Q., Mao, S. Z., Zhu, J. B., Yang, Y. S., He, H. D., Li, Y. N. 2017. Effects of grazing intensity on the ecological stoichiometry characteristics of alpine meadow. Pratacultural Science, 34(3), 449-455.

Li, H. Q., Wei, Y. X., He, H. D., Yang, Y. S., Li, Y. N. 2018. Effects of Grazing Density on Nitrous Oxide Effluxes in Alpine Kobresia Humilis Meadow on the Qinghai-Tibetan Plateau. Chinese Journal of Agrometeorology, 39(1), 27-33.

Li, H. Q., Zhang, F. W., Mao, S. J., Zhu, J. B., He, H. D., Wei, Y. X., Yang, Y. S., Li, Y. N. 2019. Effects of Grazing Density on Ecosystem CO2 Exchange of Haibei Alpine Kobresia humilis Meadow in Qinghai. Chinese Journal of Grassland, 41(2), 16-21.

Li, H. Q., Zhang, F. W., Mao, S. J., Zhu, J. B., Yang, Y. S., He, H. D., Li, Y. N. 2016. Effects of Grazing Exclusion on Soil Properties in Maqin Alpine Meadow, Tibetan Plateau, China. Polish Journal of Environmental Studies, 25(4), 1583-1587.

Li, H. Y., Yao, T., Zhang, J. G., Gao, Y. M., Yang, X. M., Li, Q., Feng, Y., Li, C. N. 2018. Temporal and Spatial Variation Characteristics of Grassland Soil Nutrients in Different Disturbed Habitats of East Qilian Mountains. Journal of Soil and Water Conservation, 32(3), 249-257.

Li, J. Y., Chu, X. H., Yin, H. Y., Mei, W. J., Xie, Y., Shan, G. L. 2021. Response of Vegetation Succession to Enclosure in *Euphorbia jolkinii* Subalpine Meadow. Chinese Journal of Grassland, 43(6), 10-16.

Li, L., He, H. D., Wei, Y. X., Yang, Y. S., Luo, J., Li, H. Q., Li, Y. N., Zhou, H. K. 2017. Response of vegetation community structure ,soil carbon sequestration ,and water-holding capacity in returning farmland to grassland plots, in the Three Rivers Source Region. Pratacultural Science, 34(10), 1999-2008.

Li, N. N. 2014. Effects of management measures on above- and below-ground biomass of plant community on alpine meadow. Lanzhou University.

Li, R. F., Niu, H. S. Kong, Q., Liu, Q. 2021. Effects of exclosure on plant and soil nutrients in an alpine grassland. Pratacultural Science, 38(3), 399-409.

Li, S. H., Gao, Q., Wang, X. Q., Lan, L., Yang, Z. W. 2016. Characteristics of Vegetation and Soil Property Changes by Photovoltaic Plant Interference in Alpine Desert Steppe. Journal of Soil and Water Conservation, 30(6), 325-329.

Li, S. Q., Wang, X. Z., Guo, Z. G., Zhou, J., Xue, R., Shen, Y. Y. 2013. Effects of Short-term Grazing on C and N Content in Soil and Soil Microbe in Alpine Meadow in the North-Eastern Edge of the Qinghai-Tibetan Plateau. Chinese Journal of Glassland, 35(1), 55-60+66.

Li, S. X., Wang, Q. J., Jing, Z. C., Wang, W. Y. 2009.The Effects of Protective Enclosure on Vegetation Diversity, and Productivity of Degraded Alpine Kobresia Meadow(Qinghai - Tibetan Plateau). Polish Journal of Ecology, 57(3), 495-502.

Li, T. C., Cao, G. M., Liu, Q. H., Zhou, G. Y., Shi, S. B., Zhang, D. G. 2012. Characteristic of four trace elements in soil and dominant plants form degraded grassland,enclosed grassland on the north bank of Qinghai Lake. Acta Prataculturae Sinica, 21(5), 213-221.

Li, W., Cao, W. X., Li, X. L., Xu, C. L., Shi, S. L. 2016. Effect of different grazing management on soil nutrient characteristics in alpine meadow-steppe. Grassland and Turf, 36(2), 8-13+20.

Li, W., Cao W. X., Liu, H. D., Li, X. L., Xu, C. L., Shi, S. L., Feng, J., Zhou, C. M. 2015. Analysis of soil respiration under different grazing management patterns in the alpine meadow-steppe of the Qinghai-Tibet Plateau. Acta Prataculturae Sinica, 24(10), 22-32.

Li, W., Cao, W. X., Shi, S. L., Li, X. L., Chen, J. G., Xu, C. L. 2016. Changes in organic carbon and nitrogen storage in alpine meadows under different grazing management regimes. Acta Prataculturae Sinica, 25(11), 25-33.

Li, W., Cao, W. X., Xu, C. L., Li, X. L., Liu, H. D., Feng, J., Shi, S. L. 2014. Changes of Vegetation Characteristics in Alpine Meadow-steppe of Eastern Qilian Mountains after Different Grazing Rest Modes. Acta Botanica Boreali-Occidentalia Sinica, 34(11), 2339-2345.

Li, W., Cao, W. X., Xu, C. L., Shi, S. L., Li, X. L., Zhang, X. J., Liu, H. D. 2015. Ecological Responses of Belowground Biomass and Soil Characteristics to Different Grazing Rest Modes in Alpine Meadow-Steppe. Acta Agrestia Sinica, 23(2), 271-276.

Li, W., Gao, W. X., Wang, G. X., Li, X. L., Xu, C. L., Shi, S. L. 2017. Effects of grazing regime on vegetation structure, productivity, soil quality, carbon and nitrogen storage of alpine meadow on the Qinghai-Tibetan Plateau. Ecological Engineering, 98, 123-133.

Li, W., Liu, Y. Z., Wang, J. L., Shi, S. L., Cao, W. X. 2018. Six years of grazing exclusion is the optimum duration in the alpine meadow-steppe of the north-eastern qinghai-tibetan plateau. Scientific Reports, 8(1).

Li, X., Li, Z. F. 2017. Characteristics of Soil Organic Carbon Content of Degraded Meadows in Napahai Plateau Wetland Region in Northwest Yunnan Province. Research of Environmental Sciences, 30(7), 1079-1088.

Li, Y., Degen, A. A., Sun, T., Wang, W. Y., Bai, Y. Y., Zhang, T. F., Long, R. J., Shang, Z. H. 2020. Three years of cultivating or fencing lands have different impacts on soil nutrients and properties of a subalpine meadow in the Tibetan Plateau. Catena, 186.

Li, Y., Yan, Z. Y., Guo, D., Wang, H. X., Su, S. L., Li, X. D., Fu, H. 2015. Effects of fencing and grazing on vegetation and soil physical and chemical properties in an alpine meadow in the Qinghai Lake Basin. Acta Prataculturae Sinica, 24(10), 33-39.

Li, Y. K., Zhang, F. W., Lin, L., Wang, X., Cao, G. M., Guo, X. W., Han, D. R., Chen, G. C. 2012. Spatiotemporal Variation in Soil Nutrient of Stipa purpurea Steppe Fenced in the Qinghai Lake Region. Chinese Journal of Applied and Environmental Biology, 18(1), 23-29.

Li, Y. T. 2017. Effects of yak grazing on CO2 flux in alpine meadow during the growing season. Lanzhou University.

Li, Y. Y., Dong, S. K., Li, X. Y., Wen, L. 2012. Effect of Enclosure on Vegetation Photosynthesis and Biomass of Degraded Grasslands in Headwater Area of Qinghai-Tibetan Plateau. Acta Agrestia Sinica, 20(4), 621-625.

Li, Y. Y., Dong, S. K., Li, X. Y., Wen, L. 2012. Effect of Grassland Enclosure on Vegetation Composition and Production in Headwater of Yellow River. Acta Agrestia Sinica, 20(2), 275-279,286.

Lin, B., Zhao, X. R., Zheng, Y., Qi, S., Liu, X. Z. 2017. Effect of grazing intensity on protozoan community, microbial biomass, and enzyme activity in an alpine meadow on the Tibetan Plateau. Journal of Soils and Sediments, 17(12), 2752-2762.

Lin, L. 2017. Response and adaptation of plant-soil system of alpine meadows in different successional stages to grazing intensity. Gansu Agricultural University.

Liu, B. 2005. Characteristics of soil element distributions of High-altitude meadow ecosystems under different Managements and degradation stages. Chengdu Institute of Biology, the Chinese Academy of Sciences, Graduate School of the Chinese Academy of Sciences, Beijing.

Liu, B. W., Jiang, Z. G. 2002. Impacts of grassland fencing on plant communities and conservation of a rare gazelle, the Przewalski's gazelle. Chinese Biodiversity, 10(3), 326-331.

Liu, J. J. 2019. Effects of the exclosure on the photosynthetic characteristics of dominant species and plant communities and simulation of productivity in alpine meadow of Qilian Mountains. Lanzhou University.

Liu, M., Zhang, Z. C., Sun, J., Wang, Y., Wang, J. N., Tsunekawa, A., Yibeltal, M., Xu, M., Chen, Y. J. 2020. One-year grazing exclusion remarkably restores degraded alpine meadow at zoige, eastern tibetan plateau. Global Ecology and Conservation, 22.

Liu, S. S. 2018. Effects of livestock and plateau zokor (*Myospalax baileyi*) on plant diversity and productivity in alpine meadow. Lanzhou University.

Liu, X. K., Zhang, Z. M., Sun, Z. H., Ou, X. K., Zhang, Y. N., Mao, Y. 2013. Impacts of different disturbances on vegetation restoration on the abandoned farmland. Ecology and Environmental Sciences, 22(6), 983-990.

Liu, X. M., Nie, X. M. 2012. Effects of enclosure on the quantitative characteristics of alpine vegetation. Pratacultural Science, 29(1), 112-116.

Liu, Y. 2018. Greenhouse gas emissions of alpine meadow grazing systems on the Qinghai-Tibet Plateau. Lanzhou University.

Liu, Y. B., Zhang, D. Y., Zhang, Y. C., Shi, M. M., Shang, Z. Y., He, L., Zong, W. J., Fu, H., Niu, D. C. 2016. Evaluation of restoration effect in degraded alpine meadow under different regulation measures. Transactions of the Chinese Society of Agricultural Engineering, 32(24), 268-275.

Liu, Y. W., Tenzintarchen, Geng, X. D., Wei, D., Dai, D. X., Xu-Ri. 2020. Grazing exclusion enhanced net ecosystem carbon uptake but decreased plant nutrient content in an alpine steppe. Catena, 195, 104799.

Liu, Y. Y., Peng, H. H., Meng, W. P., Bie, Q., Wang, Y., Zhao, C. Y. 2013. Artificial rainfall interception characteristics in alpine meadows under different grazing scenarios in the upper reach of Heihe River. Journal of Lanzhou University. Natural Science, 49(6), 799-806.

Liu, Y. Z., Cao, W. X., Wang, J. L., Li, W., Wang, S. L., Wang, X. J. 2019. Vegetation biomass allocation relationships in the different utilization patterns in alpine meadow. Grassland and Turf, 39(06), 58-65.

Liu, Z., Chen, D. D., Li, Q., Zhao, l., Xu, S. X., Zhao, X. Q. 2016. Effects of Different Land Use Patterns on Soil Inorganic Carbon in Apline Meadow Ecosystem. Bulletin of soil and Water Conservation, 36(05), 073-079.

Liu, Z. H., Wu, G. L., Yang, L. P., Banma, C. R. 2009. Benefit analysis on the returning grazing land to protected grassland in wetland protection area of Maqu in the upper reachesof Yellow River. Grassland and Turf, (3), 69-72.

Lu, H., Yao, T., Cao, L., Han, H. W. 2013. The Effect of Different Disturbed Habitat to Soil Microorganism of Alpine Grassland in Vulnerable Ecological Region. Journal of Soil Science, 44(5), 1140-1145.

Lu, H., Yao, T., Wang, D. W. 2012. Characters of vegetation in different disturbed habitats of alpine grassland in vulnerable ecological region. Grassland and Turf, 32(3), 43-48.

Lu, X., Yan, Y., Sun, J., Zhang, X., Chen, Y., Wang, X., Cheng, G. 2015. Short-term grazing exclusion has no impact on soil properties and nutrients of degraded alpine grassland in Tibet, China. Solid Earth, 6(4), 1195-1205.

Luan, J. W., Cui, L. J., Xiang, C. H., Wu, J. H., Song, H. T., Ma, Q. F., Hu, Z. D. 2014. Different grazing removal exclosures effects on soil C stocks among alpine ecosystems in east Qinghai–Tibet Plateau. Ecological Engineering, 64, 262-268.

Luo, C. Y., Wang, S. P., Zhang, L. R., Wilkes, A., Zhao, L., Zhao, X. Q., Xu, S. X., Xu, B. 2020. CO2, CH4 and N2O fluxes in an alpine meadow on theTibetan Plateau as affected by N-addition and grazing exclusion. Nutrient Cycling in Agroecosystems, 117(1), 29-42.

Luo, C. Y., Xu, G. P., Wang, Y. F., Wang, S. P., Lin, X. W., Hu, Y. G., Zhang, Z. H., Chang, X. F., Duan, J. C., Su, A. L., Zhao, X. Q. 2009. Effects of grazing and experimental warming on DOC concentrations in the soil solution on the Qinghai-Tibet Plateau. Soil Biology & Biochemistry, 41(12), 2493-2500.

Luo, L. M., Miao, Y. J., Pan, Y., Wu, J. S., Wu, J. X., Yu, C. Q., Zhao, Y., Zhao, G. F. 2015. Effects of Disturbance Intensity on the Community Characteristics and Functional Traits of Meadow Steppe at a Valley of the Lhasa River Basin. Acta Agrestia Sinica , 23(06), 1161-1166.

Ma, H. X. 2021. Effect of Different Utilization Modes and Slope Position Changes on Water Retention and Soil Consolidation in Alpine Meadows. Gansu Agricultural University.

Ma, L. N. 2014. Effects of land use and management on soil carbon and nitrogen mineralization of alpine grassland. Lanzhou University.

Ma, L. P. 2004. Study on the dynamic of soil microorganism in alpine grassland under disturbance in Tianzhu. Gansu Agricultural University.

Ma, M. J., Zhou, X. H., Du, G. Z. 2013. Effects of disturbance intensity on seasonal dynamics of alpine meadow soil seed banks on the Tibetan Plateau. Plant and Soil, 369(1-2), 283-295.

Ma, W. M., Ding, K. Y., Li, Z. W. 2016. Comparison of soil carbon and nitrogen stocks at grazing-excluded and yak grazed alpine meadow sites in Qinghai-Tibetan Plateau, China. Ecological Engineering, 87, 203-211.

Ma, Y. H. 1997. EFFECT OF GRASSLAND COMPREHENSIVE CONTROL AND MANAGEMENT IN MENGZONGGOU SMALL VALLEY, YUSHU COUNTY. Pratacultural Science, 14(5), 8-9.

Mao, S. J., Wu, Q. H., Li, H. Q., Zhang, F. W., Li, Y. N. 2015. Effects of grazing intensity on species diversity and biomass in alpine-cold forb meadow on the Tibetan Plateau. Journal of Glaciology and Geocryology, 37(5), 1372-1380.

Mao, S. J., Wu, Q. H., Zhu, J. B., Li, H. Q., Zhang, F. W., Li, Y. N. 2015. Response of the maintain performance in alpine grassland to enclosure on the Northern Tibetan Plateau. Acta Prataculturae Sinica, 24(1), 21-30.

Mei, X. M. 2017. Response and Mechanism of Soil Nematodes In Alpine Meadow to Grazing Intensity. Nanjing Agricultural University.

Miao, F. H. 2015. Response of plant community to stocking rate and precipitation variation in the grassland of northeastern edge of Qinghai-Tibetan Plateau. Lanzhou University.

Miao, F. H., Guo, Y. J., Miao, P. F., Guo, Z. G., Shen, Y. Y. 2012. Influence of enclosure on community characteristics of alpine meadow in the northeastern edge region of the Qinghai-Tibetan Plateau. Acta Prataculturae Sinica, 21(3), 11-16.

Miao, Y. J., Fu, J. J., Sun, Y. F., Chu, X. T., Yixi, C. M., Hu, T. M., Xu, Y. F. 2014. Effects of Yaks Grazing Methods on the Community Characteristics of Kobresia pygmaea Meadow in Tibet. Acta Agrestia Sinica, 22(5), 935-941.

Mipam, T. D., Chen, S., Liu, J. 2019. Short-term yak-grazing alters plant-soil stoichiometric relations in an alpine meadow on the eastern Tibetan Plateau. Plant and soil, 458, 125-137.

Mo, Q. J., Zhang, Y. F., Liu, R. T. 2008. Research on a Protection Strategy for Alpine Grassland in Gannan State by Analytic Hierarchy Process. Journal of Shanxi Agricultural Sciences, 36(6), 3-6.

Mou, X. M. 2019. Differences of soil properties effects by community and population levels in Alpine Meadow on the Qinghai-Tibetan Plateau. Lanzhou University.

Nian, Y., Ma, Y. S., Li, S. X., Wang, Y. L., Wang, X. L., Li, L. X., Qin, J. P., Zhou, X. B., Xie, L. L. 2019. Effects of summer grazing on vegetation and soil stoichiometric characteristics of Alpine marsh meadow in the upper reaches of Datong river. Chinese Qinghai Journal of Animal and Veterinary Sciences, 49(1), 6,14-18.

Nie, X. M. 2008. Study on Countermeasures and Appraisal Achievements of Grazing Withdrawal Project in Source Region of Yellow River. Gansu Agricultural University.

Niu, K. C. 2008. The Response of Reproductive Trait of Component Species to Fertilization and Grazing in Qinghai-Tibetan Alpine Meadow. Lanzhou University.

Niu, K. C., He, J. S., Zhang, S. T., Martin, J. L. 2016. Tradeoffs between forage quality and soil fertility: Lessons from Himalayan rangelands. Agriculture Ecosystems and Environment, 234, 31-39.

Niu, L., Liu, Y. H., Li, Y., Ouyang, S. N. 2015. Microbial community structure of the alpine meadow under different grazing styles in Naqu prefecture of Tibet. Chinese Journal of Applied Ecology, 26(8), 2298-2306.

Piao, S. L., Fang, J. Y. 2002. Terrestrial net primary production and its spatio-temporal patterns in Qinghai-Xizang Plateau,China during 1982-1999. Journal of Nature Resources, 17(3), 373-380.

Pu, Q., Hu, Y. F., He, J. F., Qi, P. C., Shu, X. Y., Yang, Z. P. 2016. Effect of Vegetation Restoration Pattern on the Soil Microbial Biomass and Enzyme Activity in Desertification Grassland of Northwest Sichuan. Journal of Soil and Water Conservation, 30(4), 323-328.

Qi, B. 2005. Study on the Soil Carbon Storage of Alpine Grassland under Different Dgrees of Degradation in Qinghai Lake Region. Gansu Agricultural University.

Qi, Y., Jiang, Q. O., Guo, J. B., Zhang, X. X. 2019. Effects of Seasonal Grazing on Vegetation ang Soil in Gan Nan Alpine Meadow. Acta Agrestia Sinica, 27(2), 306-314.

Qiao, C. L., Wang, J. H., Ge, S. D., Chen, D. D., Zhao, L., Li, Y. N., Xu, S. X. 2012. Comparison of soil properties under fencing and grazing in alpine meadow on QingHai-Tibet Plateau. Pratacultural Science, 29(3), 341-345.

Qiao, Y. M., Wang, Z. Q., Duan, Z. H. 2009. Effects of different land-use types on soil carbon and nitrogen contents in the northern region of Qinghai Lake. Acta Prataculturae Sinica, 18(6), 105-112.

Qin, Y. Y., Zhang, X. F., Adamowski, J. F., Biswas, A., Holden, N. M., Hu, Z. Y. 2021. Grassland grazing management altered soil properties and microbial beta-diversity but not alpha-diversity on the Qinghai-Tibetan Plateau. Applied Soil Ecology, 167.

Qiu, L. H., Qin, J. H., Zhang, Y. 2017. Effect of banning grazing on alpine meadow soil organic carbon,physio-chemical properties and enzyme activities in Binggou Watershed, Qilian Mountains. Agriculltural Research in the Arid Areas, 35(4), 179-184.

Qu, X. L., Fang, J. P. 2019. Influences of Fencing on Soil Properties and Vegetation in Degraded Shrub-grass Community. Northern Horticulture, (3), 109-115.

Ren, L., Yuan, Z. R., Chen, J. G., Li, S., Zhang, D. G., Lin, D. 2016. Characteristics of soil nutrients in alpine meadow under different utilization patterns in Eastern Qilian Mountains. Journal of Gansu Agricultural University, 51(6), 70-75.

Ren, Q., Ai, Y., Hu, J., Tian, L. M., Chen, S. Y., Minpam, T. D. 2021. Effects of different yak grazing intensities on soil and plant biomass in an alpine meadow on the Qinghai-Tibetan Plateau. Acta Ecologica Sinica, 41(17), 1-9.

Ren, Q. J., Wu, G. L., Ren, G. H. 2009. Effect of grazing intensity on characteristics of alpine meadow communities in the eastern Qinghai-Tibetan Plateau. Acta Prataculturae Sinica, 18(5), 256-261.

Ren, Y. H., Zhou, Y. Z., Jing, X. Q., Dan, Q., Ren, D. Z. 2015. Effect of Enclosure on Species Diversity and Productivity of Degraded Alpine Meadow in Tibet. Guizhou Agricultural Sciences, 43(10), 166-169.

Rui, Y. C., Wang, S. P., Xu, Z. H., Wang, Y. F., Chen, C. R., Zhou, X. Q., Kang, X. M., Lu, S. B., Hu, Y. G., Lin, Q. Y., Luo, C. Y. 2011. Warming and grazing affect soil labile carbon and nitrogen pools differently in an alpine meadow of the Qinghai-Tibet Plateau in China. Journal of Soils and Sediments, 11(6), 903-914.

Shan, G. L., Chu, X. H., Chen, G., Xie, Y., Yuan, F. J., Yin, H. Y. 2018. The Response of Soil Nutrients and Enzyme Activities to Grazing and Fencing in Sub-alpine Meadow of Northwest Yunnan. Chinese Journal of Grassland, 40(4), 82-87.

Shang, Z. H., Cao, J. J., Guo, R. Y., Henkin, Z., Ding, L. M., Long, R. J., Deng, B. 2017. Effect of enclosure on soil carbon, nitrogen and phosphorus of alpine desert rangeland. Land Degradation & Development, 28(4), 1166-1177.

Shi, F. S., Chen, H., Wu, Y., Wu, N. 2010. Effects of Livestock Exclusion on Vegetation and Soil Properties under Two Topographic Habitats in an Alpine Meadow on the Eastern Qinghai-Tibetan Plateau. POLISH JOURNAL OF ECOLOGY, 58(1), 125-133.

Shi, F. S., Wu, N., Luo, P., Luo, S. L., Wu, Y., Wang, Q., Li, Y. L., Chen, H., Gao, Y. H. 2007. Effect of Enclosuring on Community Structure of Subalpine Meadow in North-western Sichuan,China. Chinese Journal of Applied and Environmental Biology, 13(6), 767-770.

Shi, H. X., Fan, Y. J., Hou, X. Y., Yang, Y. P., Wu, X. H., Yang, T. T., Li, P. 2014. Analysis of Plant Community Characteristics of Kobresia pygmaea Meadow in the Three Headwaters under Fencing and Grazing. Chinese Journal of Grassland, 36(3), 67-72.

Shi, M. M. 2017. Effect of fencing and grazing on plant community features and functional traits in an alpine meadow. Lanzhou University.

Shi, X. M. 2011. Effects of Grassland Cultivation, *Ligularia Virgaurea* Spread and Grazing Exclusion on Soil Organic Carbon and Nitrogen Processes in Alpine Meadows. Lanzhou University.

Shi, X. M., Li, X. G., Li, C. T., Zhao, Y., Shang, Z. H., Ma, Q. F. 2013. Grazing exclusion decreases soil organic C storage at an alpine grassland of the Qinghai–Tibetan Plateau. Ecological Engineering, 57, 183-187.

Shui, H. W., Gan, Z. Z. B., Wu, H. B., Wang, Z. X., Lu, C. W., Gao, Q. Z., Hu, G. Z., Yan, J., Xie, W. D., Wang, Y. X., 2020. Effects of grazing exclusion on community characteristics and productivity of Stellera-dominated degraded grassland in the northern Tibetan Plateau. Acta Prataculturae Sinica, 29(10), 14-21.

Si, G. C., Yuan, Y. L., Wang, J., Wang, G. P., Lei, T. Z., Zhang, G. X. 2015. Effects of fencing on microbial communities and soil enzyme activities in Damxung alpine grassland. Pratacultural Science, 32(1), 1-10.

Song, L. 2016. Effects of grazing intensity and grazing system on soil physical properties and soil nutrients of alpine meadow on the northern shore of Qinghai Lake,China. Qinghai University.

Song, M. L., Wang, Y. Q., Bao, G. S., Yin, Y. L., Liu, S. C., Yang, Y. W., Yang, M., Wang, H. S. 2018. Effect of different management methods on the community structure and forage quality in Stellera-dominated degraded grassland. Pratacultural Science, 35(10), 2318-2326.

Su, S. L., Li, Y., Wang, L. Y., Guo, D., Kang, H. J., Li, X. D., Fu, H. 2014. Effect of Fencing on Plant Biomass and Functional Group Structure of Different Types of Degraded Grassland in Qinghai-Tibet Plateau. Acta Botanica Boreali-Occidentalia Sinica, 34(8), 1652-1657.

Su, S. L., Xiao, J. S., Pei, Q. S., Li, X. D., Su, W. J. 2019. Effect of grazing on vegetation characters in alpine meadow and its prediction model construction. Pratacultural Science, 36(1), 20-26.

Su, Z. S. 2015. Effects of grazing intensity on soil nutrient and vegetation community in Tibet Plateau. Northwest A & F University.

Sun, D. S. 2012. Studies on the effects of grazing intensity on vegetation and soil in alpine meadow on the eastern Qinghai-Tibetan Plateau. Lanzhou University.

Sun, G., Wu, N., Luo, P. 2005. CHARACTERISTICS OF SOIL NITROGEN AND CARBON OF PASTURES UNDER DIFFERENT MANAGEMENT IN NORTHWESTERN SICHUAN. Acta Phytoecologica Sinica, 29(2), 304-310.

Sun, G., Zhu-Barker, X., Chen, D. M., Liu, L., Zhang, N. N., Shi, C. G., He, L. P., Lei, Y. B. 2017. Responses of root exudation and nutrient cycling to grazing intensities and recovery practices in an alpine meadow: an implication for pasture management. Plant and Soil, 416(1-2), 515-525.

Sun, H. Z. 2014. Studies on the effects of Fertilization and Grazing on soil enzyme Activity in Eastern Qinghai-Tibet Plateau. Lanzhou University.

Sun, J., Peng, M., Chen, G. C., Wang, S. Z., Zhou, G. Y. 2003. Study on community characteristics and community diversity in Stipa steppe of Qinghai Lake region. Acta Botanica Boreali-Occidentalla Sinica, 23(11), 1963-1968.

Sun, J., Wang, X. D., Cheng, G. W., Wu, J. B., Hong, J. T., Niu, S. L. 2014. Effects of Grazing Regimes on Plant Traits and Soil Nutrients in an Alpine Steppe, Northern Tibetan Plateau. PLOS ONE, 9(9), e108821.

Sun, L., Wang, X. T., Wei, X. H. 2012. Effect of Closed Fencing on the Restoration of Deteriorated Alpine Grasslands in Northern Tibet Plateau. Journal of Anhui Agricultural Sciences, 40(24), 12072-12074.

Sun, L., Wang, X. T., Wei, X. H., Yang, X. M., Wu, G. L. 2012. Effects of Restroration Measures on Vegetation Features of Alpine Degraded Grassland of Amdo County in Tibet. Acta Agrestia Sinica, 20(4), 616-620.

Sun, W., Li, S. W., Wang, J. H., Fu, G. 2021. Effects of grazing on plant species and phylogenetic diversity in alpine grasslands, Northern Tibet. Ecological Engineering, 170.

Sun, Y. 2015. Interactions of soil-herbage-livestock in alpine meadow-Tibetan sheep grazing system, Qinghai-Tibetan Plateau. Lanzhou University.

Sun, Y., He, X. Z., Hou, F. J., Wang, Z. F., Chang, S. H. 2018. Grazing increases litter decomposition rate but decreases nitrogen release rate in an alpine meadow. Biogeosciences, 15(13), 4233-4243.

Tan, Y. R., Du, G. Z., Chen, D. D., Sun, D. S., Zhang, S. H., Wang, X. T. 2012. Impact of grazing on the activities of soil enzymes and soil nutrient factors in an alpine meadow on the Qinghai-Tibetan plateau. Journal of Lanzhou University. Natural Science, 48(1), 86-91.

Tenzin, T., Baima, G., Duoji, D. Z., Laba, 2018. Effects of livestock exclusion duration years on plant and soil properties in a Tibetan alpine meadow. Pratacultural Science, 5(1), 10-17.

Tserang, D. M., Wen, Y. L., Ai, Y., Zhao, H. W., Chen, Y. J. 2016. Impact of different grazing intensity on soil physical properties and plant biomass in Qinghai-Tibet Plateau alpine meadow ecosystem. Pratacultural Science, 33(10), 1975-1980.

Wang, B., Sun, G., Luo, P., Wang, M., Wu, N. 2011. Labile and recalcitrant carbon and nitrogen pools of an alpine meadow soil from the eastern Qinghai-Tibetan Plateau subjected to experimental warming and grazing. Acta Ecologica Sinica, 31(6), 1506-1514.

Wang, C. P. 2013. Effect of grazing intensity on the community structure and abundance of denitrifiers (*nirK* and *nirS*) in the Tibetan Plateau swamp meadow. Lanzhou University.

Wang, D. B. 2019. Responses of plant community and soil organic carbon to climate change and grazing in alpine meadow. Lanzhou University.

Wang, F., He, Y. T., Fu, G., Niu, B., Zhang, H. R., Li, M., Wang, Z. P., Wang, X. T., Zhang, X. Z. 2020. Effects of Enclosure on Plant and Soil Nutrients in Different Types of Alpine Grassland. Journal of Resources and Ecology, 11(03), 290-297.

Wang, H. S., Bao, G. S., Wang, Y. Q., Yin, Y. L., Yang, Y. W., Liu, S. C., Zhu, X. L., Song, M. L. 2018. Effect of different grassland managements strategies on grass productivity in Stellera-dominated degraded grasslands. Pratacultural Science, 35(11), 2561-2567.

Wang, J., Guo, N., Han, T. H., Sun, B. 2008. Ecological benefit assessment of grassland restoration projectA case study of Maqu and Anxi County in Gansu province. Pratacultural Science, 25(12), 35-40.

Wang, J., Wang, X. T., Liu, G. B., Wang, G. L., Wu, Y., Zhang, C. 2020. Fencing as an effective approach for restoration of alpine meadows: evidence from nutrient limitation of soil microbes. Geoderma, 363, 114148.

Wang, J., Wang, X. T., Liu, G. B., Wang, G. L., Zhang, C. 2021. Grazing-to-fencing conversion affects soil microbial composition, functional profiles by altering plant functional groups in a tibetan alpine meadow. Applied Soil Ecology, 166, 104008.

Wang, J. L., Li, W., Cao, W. X., Abalori, T. A., Liu, Y. Z., Xin, Y. Q., Wang, S. L., Zhang, D. G. 2021. Soil bacterial community responses to short-term grazing exclusion in a degraded alpine shrubland-grassland ecotone. Ecological Indicators, 130.

Wang, J. L., Liu, Y. Z., Cao, W. X., Li, W., Wang, X. J., Zhang, D. G., Shi, S. L., Pan, D. F., Liu, W. L. 2020. Effects of grazing exclusion on soil respiration components in an alpine meadow on the north-eastern Qinghai-Tibet Plateau. Catena, 194.

Wang, J. L., Wang, B. H., Cirenduoji, Liu, J. Z., Han, J. C. 2012. Study on improvement effects of alpine grassland at Gaize, Tibet. Prataculturae Science, 29(10), 1521-1525.

Wang, J. W, Zhao, C. Z, Zhao, L. C, Wen, J., Li, Q. 2020. Effects of grazing on the allocation of mass of soil aggregates and aggregate-associated organic carbon in an alpine meadow. PLOS ONE, 15(6), e0234477.

Wang, L., Tu, Y. L., Zhang, Y. L., Wang, Z. F., Shang, E. P. 2012. Effects of Grazing and Hydrological Disturbance on the Characteristics of Plant Community in Wet Meadow. Chinese Journal of Grassland, 34(6), 69-74.

Wang, L., Xu, X. L., Liu, L. 2014. Remotely sensed dataset of grassland degradation on the Qinghai-Tibet Plateau. Acta Geographica Sinica, 69.

Wang, L., Zhang, Y. L., Wang, Z. F., Tu, Y. L., Shang, E. P. 2013. Effects of Grazing and Hydrological Disturbance on Soil Properties of Wet Meadow Wetland in Lhasa. Research of Soil and Water Conservation, 20(1), 66-69.

Wang, L. Y. 2008. Analysis of Vegetation Variation Characteristics after Fenced Grazing in Hainan Area of Qinghai Province. Journal of Anhui Agricultural Sciences, 36(28), 12149-12150.

Wang, P., Sun, T. 2014. Effect of Rehabilitation Measures on the Soil Physical and Chemical Characteristics in the Alpine Rangeland. Research of Soil and Water Conservation, 21(4), 31-34.

Wang, Q. J., Jing, Z. C., Wang, W. Y., Lang, B. N., Ma, Y. S., Jiang, W. P. 1997. The study of grassland resource, ecological environment and sustainable deveploment in Qinghai-Xizang Plateau. Qinghai Prataculture, 6(3), 1-11.

Wang, S. Z., Fan, J. W., Li, Y. Z., Huang, L. 2019. Effects of grazing exclusion on biomass growth and species diversity among various grassland types of the tibetan plateau. Sustainability, 11(6).

Wang, W. Y., Wang, Q. J., Wang, C. Y., Shi, H. L., Li, Y., Wang, G. 2005. The effect of land management on carbon and nitrogen status in plants and soils of alpine meadows on the Tibetan plateau. Land Degradation & Development, 16(5), 405-415.

Wang, W. Y., Wang, Q. J., Wang, G., Jing, Z. C. 2007. EFFECTS OF LAND DEGRADATION AND REHABILITATION ON VEGETATION CARBON AND NITROGEN CONTENT OF ALPINE MEADOW IN CHINA. Journal of Plant Ecology, 31(6), 1073-1078.

Wang, W. Y., Zeng, Z. K., Yin, H. X., Chen, K. H. 2009. Effects of different land management measures on vegetation productivity on alpine Kobresia meadow. Journal of Lanzhou University. Natural Science, 45(2), 43-47.

Wang, X. D., Yan, Y., Cao, Y. Z. 2012. Impact of historic grazing on steppe soils on the northern Tibetan Plateau. Plant and Soil, 354(1-2), 173-183.

Wang, X. L., Gan, Y. M., Zhang, L., Zhang, D. G., Zhou, X. H., Miao, X. L., Deng, C. H., Qi, B., Yang, Y. H., Guan, Q. Z. X. 2005. Influence of fencing and light grazing on enclosed alpine steppes in the Qinghai lake area. Journal of Gansu Agricultural University, 40(3), 368-375.

Wang, X. M. 2014. Effects of carbon and nitrogen additions on the mineralization of Soil carbon, nitrogen, and Nitrogen Transformation Rate of Alpine Meadow in Northern Tibet. Lanzhou University.

Wang, X. T. 2010. Effects of Different Grazing Intensities o Vegetation and Soil Physical and Chemcial Character in Alpine Meadow. Lanzhou University.

Wang, X. X., Dong, S. K., Li, Y. Y., Li, X. Y., Wen, L., Wu, Y. 2012. Effects of Grassland Degradation and Artificial Restoration on Soil Physicochemical Properties in Three-river Headwater. Journal of Soil and Water Conservation, 26(4), 113-117,122.

Wang, Y. B. 2017. The influence of short-term fencing to the vegetation quantity characteristics of degraded alpine meadow and soil nutrient. Qinghai Prataculture, 26(4), 14-17.

Wang, Y. J., Wei, X. H., Yang, P. 2005. Effects of over-grazing on vegetation degradation of *Kobresia pygmaea* meadow in Naqu, Tibet. Journal of Lanzhou University (Natural Sciences), 41(1), 32-38.

Wang, Y. L., Ma, Y. S., Dong, Q. M., Shi, J. J., Liu, D. M., Li, S. X., Yang, S. H., Sheng, L. 2011. Effect of Forbidden Pasturing on Biomass of Different Degraded Degree Grassland in Area of Yollow River Source. Chinese Qinghai Journal of Animal and Veterinary Sciences, 41(3), 14-16.

Wang, Y. Q., Bao, G. S., Song, M. L., Yin, Y. L., Liu, S. C., Yang, Y. W., Yang, M., Wang, H. S. 2018. Effects of nitrogen fertilizer on community structure and nutritional quality of degraded grassland of Stellera chamaejasme under two different management measures. Acta Prataculturae Sinica, 27(12), 177-186.

Wang, Y. Q., Bao, G. S., Wang, H. S., Yin, Y. L., Song, M. L., Zeng, H., Ma, G. L., Luo, Z. A. M. 2016. Effect of burrowing activity of *Myospalax baileyi* on soil physical – chemical properties in different grazing systems. Chinese Qinghai Journal of Animal and Veterinary Sciences, 46(6), 5-10.

Wang, Y. Q., Bao, G. S., Wang, H. S., Zeng, H., Li, J. P. 2018. Effect of Burrowing Activity of Myospalaxbaileyi on Plant Community Structure in Different Grazing Systems. Acta Agrestia Sinica, 26(1), 134-141.

Wang, Y. X. 2020. Responses of the structure and function of alpine ecosystem to plant-livestock interactions. Lanzhou University.

Wang, Y. X., Hodgkinson, K. C., Hou, F. J., Wang, Z. F., Chang, S. H. 2018. An evaluation of government‐recommended stocking systems for sustaining pastoral businesses and ecosystems of the alpine meadows of the qinghai‐tibetan plateau. Ecology and Evolution, 8(8), 4252-4264.

Wang, Z., Luo, T. X., Li, R. C., Tang, Y. H., Du, M. Y. 2013. Causes for the unimodal pattern of biomass and productivity in alpine grasslands along a large altitudinal gradient in semi-arid regions. Journal of Vegetation Science, 24(1), 189-201.

Wang, Z. Q. 2009. Effects of different land-use types and vegetation degradation on soil carbon and nitrogen content of the Alpine Steppes in the northern region of the Qinghai Lake. Qinghai University.

Wei, D., Xu, R., Wang, Y. H., Wang, Y. S., Liu, Y. W., Yao, T. D. 2012. Responses of CO2, CH4 and N2O fluxes to livestock exclosure in an alpine steppe on the Tibetan Plateau, China. Plant and Soil, 359(1-2), 45-55.

Wei, Y. L. 2019. Response mechanism of soil microorganisms to livestock grazing in alpine grassland. Qinghai Normal University.

Wei, Y. L., Cao, W. X., Li, J. H., Zhang, A. M., Li, X. L. 2018. Phospholipidfatty acid (PLFA) analysis of soil microbial community structure with different intensities of grazing and fencing in alpine shrubland. Acta Ecologica Sinica, 38(13), 4897-4908.

Wei, Y. L., Cao, W. X., Liu, Y. Z. 2018. Effect of grazing intensity and fencing on soil microbial biomass in alpine shrubland. Glassland and Turf, 38(5), 1-7.

Wu, G. L., Du, G. Z., Liu, Z. H., Thirgood, S. 2009. Effect of fencing and grazing on a Kobresia-dominated meadow in the Qinghai-Tibetan Plateau. Plant and Soil, 319(1-2), 115-126.

Wu, G. L., Li, W., Zhao, L. P., Shi, Z. H. 2011. Artificial ManagementImproves Soil Moisture, C, N and P in an Alpine Sandy Meadow of Western China. Pedosphere, 21(03), 407-412.

Wu, G. L., Liu, Z. H., Lei, Z., Chen, J. M., Hu, T. M. 2010. Long-term fencing improved soil properties and soil organic carbon storage in an alpine swamp meadow of western China. Plant and Soil, 332(1-2), 331-337.

Wu, H. Y., Ma, Y. S., Dong, Q. M., Sun, X. D., Shi, J. J., Wang, Y. L., Sheng, L. 2009. Seasonal dynamics of aboveground biomass and nutrients of Kobresia tibetica meadow in Yellow River headwater area. Prataculturae Science, 26(1), 8-12.

Wu, J. B., Wang, X. D. 2017. Effect of Enclosure Ages on Community Characters and Biomass of the Degraded Alpine Steppe at the Northern Tibet. Acta Agrestia Sinica, 25(2), 261-266.

Wu, J. S., Zhang, X. Z., Shen, Z. X., Shi, P. L., Xu, X. L., Li, X. J. 2013. Grazing-exclusion effects on aboveground biomass and water-use efficiency of alpine grasslands on the northern tibetan plateau. Rangeland Ecology & Management, 66(4), 454-461.

Wu, Q. H., Mao, S. J., Liu, X. Q., Li, H. Q., Zhang, F. W., Li, Y. N. 2014. Analysis of the soil water-holding capacity in alpine forb meadow under grazing gradient and relevant influence factors. Journal of Glaciology and Geocryology, 36(3), 590-598.

Wu, R. X. 2015. Growth and reproductive characteristics of *Thermopsis lanceolata* and its responses to management practices in alpine meadow of the Qinghai-Tibetan Plateau. Gansu Agricultural University.

Wu, S. M., Liu, Y. H., Li, Y., Nie, C., Du, W. 2017. Effect of temperature and humidity on the alpine meadow soil carbon mineralization under different management in Naqu prefecture in Tibet. Journal of Beijing Normal University. Natural Science, 53(5), 615-623.

Wu, X. W., Wang, Y. C., Sun, S. C. 2019. Long-term fencing decreases plant diversity and soil organic carbon concentration of the Zoige alpine meadows on the eastern Tibetan plateau. Plant and Soil, 458(1-2), 191-200.

Xiang, M. X., La, D., Wu, J. X., Wu, J. J. 2019. Response of plant community characteristics and soil nutrients to different grazing intensities in the Lhasa River Valley. Plateau Science Research, 3(4), 32-39.

Xiang, M. X., Wu, J. X., Wu, J. J., Guo, Y. J., Lha, D., Pan, Y., Zhang, X. Z. 2021. Heavy Grazing Altered the Biodiversity-Productivity Relationship of Alpine Grasslands in Lhasa River Valley, Tibet. Frontiers in Ecology and Evolution, 9.

Xiang, T. Z. M. 2011. Analysis the effect on biomass in closed grassland. Qinghai Prataculture, 20(3), 11-12.

Xiao, R. 2012. Effects of long-term fertilization on the community structure diversity of prokaryotic microbes and ammonia oxidizers in the Alpine Meadow soil on Tibet Plateau. Lanzhou University.

Xie, Y., Yin, H. Y., Chu, X. H., Yuan, F. J., Chen, G., Shan, G. L. 2017. Effects of Grazing and Fencing on Soil Microbial Characteristics in Sub-alpine Meadow of Northwest Yunnan. Journal of Yunnan Agricultural University, 32(6), 1121-1128.

Xiong, D. P., Shi, P. L., Sun, Y.L, Wu, J. S., Zhang, X. Z. 2014. Effects of Grazing Exclusion on Plant Productivity and Soil Carbon, Nitrogen Storage in Alpine Meadows in Northern Tibet, China. Chinese Geographical Science, 24(4), 488-498.

Xu, T. W., Zhao J. C., Mao, S. J., Geng, Y. Y., Liu, H. J., Zhao, X. Q., Xu, S. X. 2020. Response of plant community structure and biomass to short-term rest grazing in an alpine meadow in Haibei Autonomous Prefecture of Qinghai. Acta Prataculturae Sinica, 29(4), 1-8.

Xu, X. B., Cao, J. J., Yang, L., Yang, S. R., Gong, Y. F., Li, M. T. 2018. Effects of grazing and enclosure on foliar and soil stoichiometry of grassland on the Qing-hai-Tibetan Plateau. Chinese Journal of Ecology, 37(5), 1349-1355.

Xu, Y. F., Yixicuomu, Fu, J. J., Chen, H., Miao, Y. J., Chen, J., Hu, T. M., Shan, J. G. 2012. Response of Plant Diversity and Soil Nutrient to Grazing Intensity in Kobresia pygmaea Meadow of Qinghai-Tibet Plateau. Acta Agrestia Sinica, 20(6), 1026-1032.

Xu, Z. R., Lu, C. X., Cheng, S. K., Bijaya, G. C. D. 2016. Effect of pasture enclosure and dung droppings on soil nutrients and aboveground biomass in alpine grassland in the Northern Tibetan Plateau. The Journal of Animal & Plant Sciences, 26(5), 1361-1367.

Xue, H. Y., Luo, D. Q., Hu, F., Li, H. X., Wang, J. S., Qu, X. L., Wang, H. Y., Yu, B. Z., Sun, Q. 2016. Effect of short-term enclosure on soil nematode communities in an alpine meadow in Northern Tibet. Acta Ecologica Sinica, 36(19), 6139-6148.

Xue, Y. F., Zong, N., He, N. P., Tian, J., Zhang, Y. Q. 2018. Influence of long-term enclosure and free grazing on soil microbial community structure and carbon metabolic diversity of alpine meadow. Chinese Journal of Applied Ecology, 29(8), 2705-2712.

Yan, S. Y., Zhou, Z. Y., Qin, H., Zhou, L. N. 2010. Characteristics of nitrogen contents under different land use conditions in Alpine grassland of Maqu. Acta Prataculturae Sinica, 19(2), 153-159.

Yan, X. 2021. Mechanisms and interaction effects of short-term nitrogen and phosphorus fertilization and cold-season grazing on plant diversity and productivity of alpine meadow. Lanzhou University.

Yan, Y., Lu, X. Y. 2015. Is grazing exclusion effective in restoring vegetation in degraded alpine grasslands in Tibet, China? PeerJ, 3.

Yan, Y., Ma, X. X., Lu, X. Y. 2014. Effect of Human Disturbance on Plant Biomass and CNP Contents of the Alpine Steppe in Northern Tibet. Mountain Research, 32(04), 31-460-466.

Yang, C. M., Xie, Y., Chu, X. H., Chen, G., Yuan, F. J., Yin, H. Y., Shan, G. L. 2018. Effects of Different Disturbing Approaches on Biomass and Carbon Storage of Sub-alpine Meadow in Northwest Yunnan Province. Chinese Journal of Grassland, 40(3), 62-67.

Yang, C. T., Zhang, Y., Hou, F. J., Millner, J. P., Wang, Z. F., Chang, S. H. 2019. Grazing activity increases decomposition of yak dung and litter in an alpine meadow on the Qinghai-Tibet plateau. Plant and Soil, 444(1-2), 239-250.

Yang, F., Niu, K. C., Collins, C. G., Yan, X. B., Ji, Y. G., Ling, N., Zhou, X. H., Du, G. Z., Guo, H., Hu, S. J. 2018. Grazing practices affect the soil microbial community composition in a Tibetan alpine meadow. Land Degradation and Development, 30(1), 49-59.

Yang, J. 2021. Effects of Grazing and Disturbance of Plateau Zokors on Reproductive Characteristics of *Potentilla Anserine*. Gansu Agricultural University.

Yang, J. Q., Yuan, M. M., Zhang, X. Y., Zhang, Z. W., Si, W. T., Shang, Z. H. 2010. The effects of grazing prohibition on grassland at Longbao national nature reserve. Grassland and Turf, 30(5), 46-49.

Yang, Q., He, G. Y., Sun, H. Z., Du, G. Z. 2013. The response of soil physico-chemcial property and microbial biomass to grazing on Tibetan Plateau. Journal of Gansu Agricultural University, 48(4), 76-81.

Yang, Y. D. 2021. Effects of Different Enclosure Years on Soil Physicochemical Properties and Microorganisms in Degraded Grassland. Grassland and Prataculture, 33(1), 41-46.

Yang, Z. A. 2017. A STUDY ON THE RESPONSES OF VEFETATION-SOIL SYSTEM TO GRAZING AND NITROGEN ADDITION IN AN ALPINE MEADOW OF QINGHAI-TIBETAN PLATEAU. Northwest A&F University.

Yang, Z. A., Jiang, L., Xu, Y. Y., Zhan, W., Zhu, E. X., Chen, H. 2017. Responses of vegetation and soil of alpine meadows on the Qinghai-Tibet Plateau to short-term grazing prohibition. Acta Ecologica Sinica, 37(23), 7903-7911.

Yang, Z. A., Xiong, W., Xu, Y. Y., Jiang, L., Zhu, E. X., Zhan, W., He, Y. X., Zhu, D., Zhu, Q. A., Peng, C. H., Chen, H. 2016. Soil properties and species composition under different grazing intensity in an alpine meadow on the eastern Tibetan Plateau, China. Environmental Monitoring and Assessment, 188(12).

Yang, Z. L., Hautier, Y., Borer, E. T., Zhang, C. H., Du, G. Z. 2015. Abundance- and functional-based mechanisms of plant diversity loss with fertilization in the presence and absence of herbivores. Oecologia, 179(1), 261-270.

Yao, T., Wang, G., Zhang, D. G., Long, R. J. 2006. Temporal changes of grassland vegetation, soil and soil microbial population in the Tianzhu alpine region. Acta Ecologica Sinica, 26(6), 1926-1932.

Yao, X. X. 2019. Studies on response mechanism of plant community and livestock to grazing pressure in an alpine meadow. Gansu Agricultural University.

Yao, X. X., Gong, X. Y., Zhang, L. P., Jiao, T., Tao, H. X., Guo, B., Zhang, A. Q., Wu, J. P. 2018. Effects of Grazing and Long-term Fencing on Nutritive Values of Dominant Species in Alpine Meadow of Qilian Mountains. Acta Agrestia Sinica, 26(6), 1354-1362.

Yao, X. X., Gong, X. Y., Zhang, L. P., Wang, J. F., Lang, X., Wang, C. L., Song, S. Z., Wu, J. P. 2018. Effects of Different Grazing Intensities on Nutritive Values of Dominant Species in Alpine Meadow of Qilian Mountains. Acta Agrestia Sinica, 26(5), 1159-1167.

Yao, X. X., Wu, J. P., Gong, X. Y., Lang, X., Wang, C. L., Song, X. Z., Ahmad, A. A. 2019. Effects of long term fencing on biomass, coverage, density, biodiversity and nutritional values of vegetation community in an alpine meadow of the Qnghai-Tbet Pateau. Ecological Engineering, 130, 80-93.

Yi, X. C. M., Xu, Y. F., Fu, J. J., Sun, Y. F., Ba, S. J. B., Nibu, Hu, T. M., Miao, Y. J. 2014. Effects of grazing intensity on vegetation community and soilphysicochemical properties of alpine meadow in Tibet. Journal of Northwest A & F University. Natural Science Edition, 42(6), 27-33.

Yin, Y. L., Wang, Y. Q., Li, S. X., Liu, Y., Zhao, W., Ma, Y. S., Bao, G. S. 2019. Effects of enclosing on soil microbial community diversity and soil stoichiometric characteristics in a degraded alpine meadow. Chinese Journal of Applied Ecology, 30(1), 127-136.

Yin, Y. L., Wang, Y. Q., Li, S. X., Liu, Y., Zhao, W., Ma, Y. S., Bao, G. S. 2021. Soil microbial character response to plant community variation after grazing prohibition for 10 years in a Qinghai-Tibetan alpine meadow. Plant and Soil, 458(1-2), 175-189.

Yu, B. Z., Peng, Y. L., Qu, X. L. 2019. Impact of Short-term Enclosure on Soil Organic Carbon and Main Nutrients in Alpine Meadow. Southwest China Journal of Agricultural Sciences, 32(5), 1074-1078.

Yu, H. 2013. Dynamics of Grassland Growth and Its Response to Climate Change on Tibetan Plateau. Lanzhou University.

Yu, L. F., Chen, Y., Sun, W. J., Huang, Y. 2019. Effects of grazing exclusion on soil carbon dynamics in alpine grasslands of the Tibetan Plateau. Geoderma, 353, 133-143.

Yu, X. J., Jing, Y. Y., Duan, C. H., Xu, C. L., Yang, H. L., Luo, J. L., An, Y. F., An, X. D. 2015. Influence of enclosure and grazing intensity on alpine meadow vegetation and soil characteristics in the Easter Qilian Mountains. Agriculltural Research in the Arid Areas, 33(1), 252-257+277.

Yuana, Z. Q., Epstein, H., Lia, G. Y. 2020. Grazing exclusion did not affect soil properties in alpine meadows in the tibetan permafrost region. Ecological Engineering, 147(23), 105657.

Yuana, Z. Q., Jiang, X. J. 2021. Vegetation and soil covariation, not grazing exclusion, control soil organic carbon and nitrogen in density fractions of alpine meadows in a tibetan permafrost region. Catena, 196.

Zhai, W. T., Chen, D. D., Li, Q., Zhao, L., Liu, Z., Xu, S. X., Dong, Q. M., Zhao, X. Q. 2017. Effect of grazing intensity on carbon metabolic characteristics of soil microbial communities in an alpine steppe in the regions around Qinghai Lake. Chinese Journal of Applied and Environmental Biology, 23(4), 685-692.

Zhan, W. 2015. Grazing effects on nitrous oxide emissions of alpine meadow in Tibet Plateau. Northwest A & F University.

Zhang, D. Y. 2015. Responses of plant functional traits and community assembly to different disturbances in alpine meadoe. Lanzhou University.

Zhang, F., Qi, B., Wen, F., Zhang, D. G., Wu, H., Zhang, L. 2011. Analysis of the change of carbon storage in alpine arid grassland. Acta Prataculturae Sinica, 20(4), 11-18.

Zhang, G. R., Li, H. Q., Yang, Y. S., Wang, J. B., Zhu, J. B., Luo, J., He, H. D., Li, Y. N. 2020. Comprehensive Evaluation of Grassland Quality under Different Restoration Methods in Degraded Alpine Medaow Based on Principal Component Analysis. Chinese Journal of Grassland, 42(2), 76-82.

Zhang, J. 2016. Effect of Grazing on Carbon and Nitrogen Reserve of Tibet Alpine Typical Wetland. Hubei Agricultural Sciences, 55(18), 4660-4663.

Zhang, H. 2017. Effects of different land use types and fertilization on soil organic carbon storages in alpine meadows. Lanzhou University.

Zhang, H., He, Q. F., Pandey, S. P., Jiang, K., Wang, C. 2021. Can Overgrazing Responses Be Disentangled by Above- and Below-Ground Traits? Frontiers in Ecology and Evolution, 9.

Zhang, J. S. 2020. Effects of grazing exclusion on plant community composition and carbon storage of alpine meadow in Qinghai-Tibet Plateau. Lanzhou University.

Zhang, L., Wang, J., Wang, X. T., Liao, L. R., Wan, Q., Liu, G. B., Zhang, C. 2021. Effect of restoration types on the community structure of microbes harboring *nifH* and *chiA* genes in alpine meadow. Chinese Journal of Applied Ecology, 1-11.

Zhang, Q. 2019. The Assessment of Natural and Anthropogenic Relative Contribution to Grassland Degradation of the Qinghai-Tibet Platea. Northwest A & F University.

Zhang, Q., Wang, Z. C., Pu, Q. S., Hou, Q. Q., Cai, Z. Y., Yang, J., Yao, B. H., Wang, C., Sun, X. M., Su, J. H. 2020. Effects of Different Management Modes on Carbon Storage in Gannan Alpine Meadow. Acta Agrestia Sinica, 28(02), 529-537.

Zhang, Q., Yang, J., Yao, B. H., Cai, Z. Y., Sun, X. M., Wang, C., Guo, H. L., Tan, Y. C., Su, J. H. 2020. Effects of different grazing strategies on zokor mounds plant community succession in alpine meadow. Acta Ecologica Sinica, 40(8), 2802-2811.

Zhang, Q., Yang, J., Yao, B. H., Cai, Z. Y., Wang, X. Y., Su, J. H. 2021. Effects of grazing modes on soil physical, chemical properties and species diversity in alpine meadow in the eastern margin of Qilian Mountains. Grassland and Turf, 41(2), 105-112.

Zhang, T. 2016. Effects of carbon and nitrogen addition, Fencing and Mowing on soil and vegetation of alpine meadow in Northern Tibet. Lanzhou University.

Zhang, T., Zhang, Y. J., Xu, M. J., Zhu, J. T., Wimberly, M. C., Yu, G. R, Niu, S. L., Xi, Y., Zhang, X. Z., Wang, J. S., 2015. Light-intensity grazing improves alpine meadow productivity and adaption to climate change on the Tibetan Plateau. Scientific Reports, 5, 15949.

Zhang, W. N. 2015. The Plant and Soil Characteristics of the Alpine Meadow under Different Period of Grazing Ban in Northern Tibet. Chinese Academy of Agricultural Sciences Dissertation.

Zhang, W. N., Ganjurjav, Li, Y. W., Gao, Q. Z., Wan, Y. F., Li, Y. E., Danjiu, L. B., Xirao, Z. M., Baima, Y. Z. 2013. Effects of Banning Grazing and Delaying Grazing on Species Diversity and Biomass of Alpine Meadow in Northern Tibet. Journal of Agricultural Science and Technology, 15(3), 143-149.

Zhang, W. N., Ganjurjav, H., Liang, Y., Gao, Q. Z., Wan,Y. F., Li, Y., Baima, Y. Z., Xirao, Z. M. 2015. Effect of a grazing ban on restoring the degraded alpine meadows of Northern Tibet, China. The Rangeland Journal, 37(1), 89-95.

Zhang, Y. 2016. Effects of summer and winter rotational grazing of Tibetan sheep on plant, soil and livestock of alpine ecosystem. Lanzhou University.

Zhang, Y., Gao, Q. Z., Ganjurjav, H., Dong, S. K., Zheng, Q. Z., Ma, Y. D., Liang, K. M. 2021. Grazing Exclusion Changed the Complexity and Keystone Species of Alpine Meadows on the Qinghai-Tibetan Plateau. Frontiers in Ecology and Evolution, 9.

Zhang, Y. C., Yuan, X. B., Niu, D. C., Wu, S. J., Zhang, D. Y., Zong, W. J., Fu, H. 2016. Response of plateau pika burrow density to vegetation management in an alpine meadow,Maqu County ,Gansu. Acta Prataculturae Sinica, 25(2), 87-94.

Zhang, Y. X., Yao, T., Wang, G. J., Ma, W. W., Ma, W. B. 2014. Characteristics of vegetation and soil inorganic nitrogen concentrations under different disturbed habitats in a weak alpine ecosystem. Acta Prataculturae Sinica, 23(4), 245-252.

Zhang, Z., Duo, H. R., Yang, M., Zhou, Y., Lv, S., Wen, L., Lei, G. C. 2018. Ecosystem Respiration of Alpine Steppe and Alpine Meadow in Riparian Zones of Qinghai Lake and Yellow River Source Region under Different Grazing Ways. Wetland Science, 16(2), 251-258.

Zhao, B. B., Niu, K. C., Du, G. Z. 2009. The effect of grazing on above-ground biomass allocation of 27 plant species in an alpine meadow plant community in Qinghai-Tibetan Plateau. Acta Ecologica Sinica, 29(3), 1596-1606.

Zhao, J. X., Li, X., Li, R. C., Tian, L. H., Zhang, T. 2016. Effect of grazing exclusion on ecosystem respiration among three different alpine grasslands on the central Tibetan Plateau. Ecological Engineering, 94, 599-607.

Zhao, J. X., Luo, T. X., Li, R. C., Li, X., Tian, L. H. 2016. Grazing effect on growing season ecosystem respiration and its temperature sensitivity in alpine grasslands along a large altitudinal gradient on the central tibetan plateau. Agricultural and Forest Meteorology, 218, 114-121.

Zhao, J. X., Qi, B., Duo, J. D. Z., Shang, Z. H. 2011. Effects of short-term enclose on the community characteristics of three types of degraded alpine grasslands in the north Tibet. Prataculturae Science, 28(01), 59-62.

Zhao, J. X., Sun, F. D., Tian, L. H. 2018. Altitudinal pattern of grazing exclusion effects on vegetation characteristics and soil properties in alpine grasslands on the central Tibetan Plateau. Journal of Soils and Sediments, 19(2), 750-761.

Zhen, W., Dong, Q. M., Li, S. X., Shi, J. J., Liu, Y., Hou, X. K., Song, L. 2013. Dynamics of plants community of alpine steppe under enclosure around Qinghai Lake. Prataculturae Science, 31(06), 1126-1130.

Zhen, W., Li, S. X., Dong, Q. M., Liu, Y. 2013. Effects of Grazing Systems on the Community Characteristics of Alpine Steppe in Qinghai Lake Region. Acta Agrestia Sinica, 21(5), 869-874.

Zhou, G. L., Cheng, Y. X., Ma, Q. Q., Shen, B., Qu, J., Tian, F., Chang, S. H. 2019. Effects of grazing intensity on community structure and the soil's physical and chemical properties in an alpine meadow on the Eastern Qinghai-Tibet Plateau. Pratacultural Science, 36(4), 1022-1031.

Zhou, G. Y., Chen, G. C, Xu, W. H., Yang, L. C., Han, Y. J., Li, J. P. 2010. Influences of enclosure to Achnatherum splendens steppes biomass in the Qinghai Lake Area. Arid Land Geography, 33(3), 434-441.

Zhou, G. Y., Chen, G. C., Zhao, Y. L., Wang, S. Z., Sun, J. 2005. Comparative studies on the influence of chemical fertilizer application and enclosure on alpine steppes in Qinghai Lake area ⅡSeasonal and Annual Biomass Dynamics. Pratacultural Science, 22(1), 59-63.

Zhou, H. K., Tang, Y. H., Zhao, X. Q., Zhou, L. 2006. Long-term grazing alters species composition and biomass of a shrub meadow on the Qinghai-Tibet Plateau. Pakistan Journal of Botany, 38(4), 1055-1069.

Zhou, T. Y., Gao, J., Wang, J. N., Sun, J., Xu, B., Xue, J. Y., He, J. D., Xie, Y., Wu, Y. 2018. Effects of 7-years enclosure on an alpine meadow at the south-eastern margin of Tibetan Plateau based on community structure and soil physical-chemical properties. Acta Prataculturae sinica, 27(12), 1-11.

Zhou, W. C., Suolang, Duoerji, Cui, L. J., Wang, Y. F., Li, W. 2015. Effects of Fencing and Grazing on the Emissions of CO2 and CH4 in Zoige Peatland, East Qinghai-Tibetan Plateau. Ecology and Environmental Sciences, 24(2), 183-189.

Zhou, X. Y. 2019. Impacts of different restoration years of returning grazing land to grassland on community characteristics and soil nutrients of alpine grassland in Maqu County. Northwest Normal University.

Zhu, G. Q., Yuan, C. X., Gong, H. D., Peng, Y. L., Huang, C. J., Wu, C. A. S., Duan, H. C. 2021. Effects of short-term grazing prohibition on soil physical and chemical properties of meadows in Southwest China. PeerJ, 9.

Zhu, J. B., He, H. D., Li, H. Q., Yang, Y. S., Wei, Y. X., Luo, J., Li, Y. N. 2018. The Response of Soil Water Storage to Different Grazing Gradients in An Alpine Meadow. Chinese Journal of Grassland, 40(4), 88-94.

Zhu, J. T., Zhang, Y. J., Liu, Y. J. 2016. Effects of short-term grazing exclusion on plant phenology and reproductive succession in a Tibetan alpine meadow. Scientific Reports, 6.

Zhu, L., Zhao, M., Li, G. Y., Chen, W. Y., Wei, Q., Wang, F., Zhang, J. Q., Kang, J. J., Bing, D. H. 2013. Effects of Enclosure on Revegetation of Degradation Swamp Wetland in Hequ Stud-farm. Chinese Agricultural Science Bulletin, 29(16), 54-57.

Zhu, W. X. 2012. The impact of grassland, grazing on soil microbial biomass on the eastern Tibetan Plateau. Lanzhou University.

Zhu, X. H., Xie, Y., Shan, G. L., Yuan, F. J., Chen, G., Yin, H. Y. 2017. Effect of Management Patterns on Community Structure and Species Diversity of Subalpine Meadow of the Southern Margin of Tibetan Plateau. Acta Agrestia Sinica, 25(5), 939-945.

Zong, N., Shi, P. L., Jiang, J., Meng, F. S., Ma, W. L., Xiong, D. P., Song, M. H., Zhang, X. Z. 2013. Effects of Fertilization and Grazing Exclosure on Vegetation Recovery in a Degraded Alpine Meadow on the Tibetan Plateau. Chinese Journal of Applied and Environmental Biology, 19(6), 905-913.

Zong, N., Shi, P. L., Jiang, Q., Xiong, D. P., Meng, F. S., Song, M. H., Zhang, X. Z., Shen, Z. X. 2013. Interactive effects of short-term nitrogen enrichment and simulated grazing on ecosystem respiration in an alpine meadow on the Tibetan Plateau. Acta Ecologica Sinica, 33(19), 6191-6201.

Zong, N., Shi, P. L., Zheng, L. L., Zhou, T. C., Cong, N., Hou, G., Song, M. H., Tian, J., Zhang, X. Z., Zhu, J. T. 2021. Restoration effects of fertilization and grazing exclusion on different degraded alpine grasslands: Evidence from a 10-year experiment. Ecological Engineering, 170.

Zou, J., Zhao, L., Xu, S., Xu, X., Chen, D., Li, Q., Zhao, N., Luo, C., Zhao, X. 2014. Field (CO2)-C-13 pulse labeling reveals differential partitioning patterns of photoassimilated carbon in response to livestock exclosure in a Kobresia meadow. Biogeosciences, 11(16), 4381-4391.

Zou, J. R., Luo, C. Y., Xu, X. L., Zhao, N., Zhao, L., Zhao, X. Q. 2016. Relationship of plant diversity with litter and soil available nitrogen in an alpine meadow under a 9-year grazing exclusion. Ecological Research, 31(6), 841-851.

**Table S2** The effects of annual mean temperature (AMT) and annual mean precipitation (AMP) on ΔCSFI in different grazing exclusion (GE) duration. Explanation degree (*R*^2^) and significant level (*P*) were estimated by general linear model (GLM).

| Ecosystem  type | GE duration  (year) |  | AMP | |  | AMT | |
| --- | --- | --- | --- | --- | --- | --- | --- |
|  |  |  | *R*^2^ | *P* |  | *R*^2^ | *P* |
| Alpine  meadow | 1 |  | 0.05 | 0.34 |  | 0.03 | 0.44 |
|  | 2 |  | 0.04 | 0.27 |  | 0.02 | 0.44 |
|  | 3 |  | 0.001 | 0.87 |  | 0.03 | 0.38 |
|  | 4 |  | 0.24 | 0.003 |  | 0.06 | 0.16 |
|  | 5 |  | 0.38 | 0.04 |  | 0.38 | 0.04 |
|  | 6 |  | 0.34 | 0.001 |  | 0.13 | 0.05 |
|  | 7 |  | 0.02 | 0.41 |  | 0.04 | 0.23 |
|  | 8 |  | 0.03 | 0.53 |  | 0.004 | 0.82 |
|  | 9 |  | 0.34 | 0.002 |  | 0.40 | 0.001 |
|  | 10 |  | 0.33 | 0.005 |  | 0.28 | 0.01 |
|  | 11 |  | 0.61 | 0.003 |  | 0.56 | 0.005 |
| Alpine  steppe | 2 |  | 0.35 | 0.29 |  | 0.35 | 0.29 |
|  | 3 |  | 0.60 | 0.07 |  | 0.60 | 0.07 |


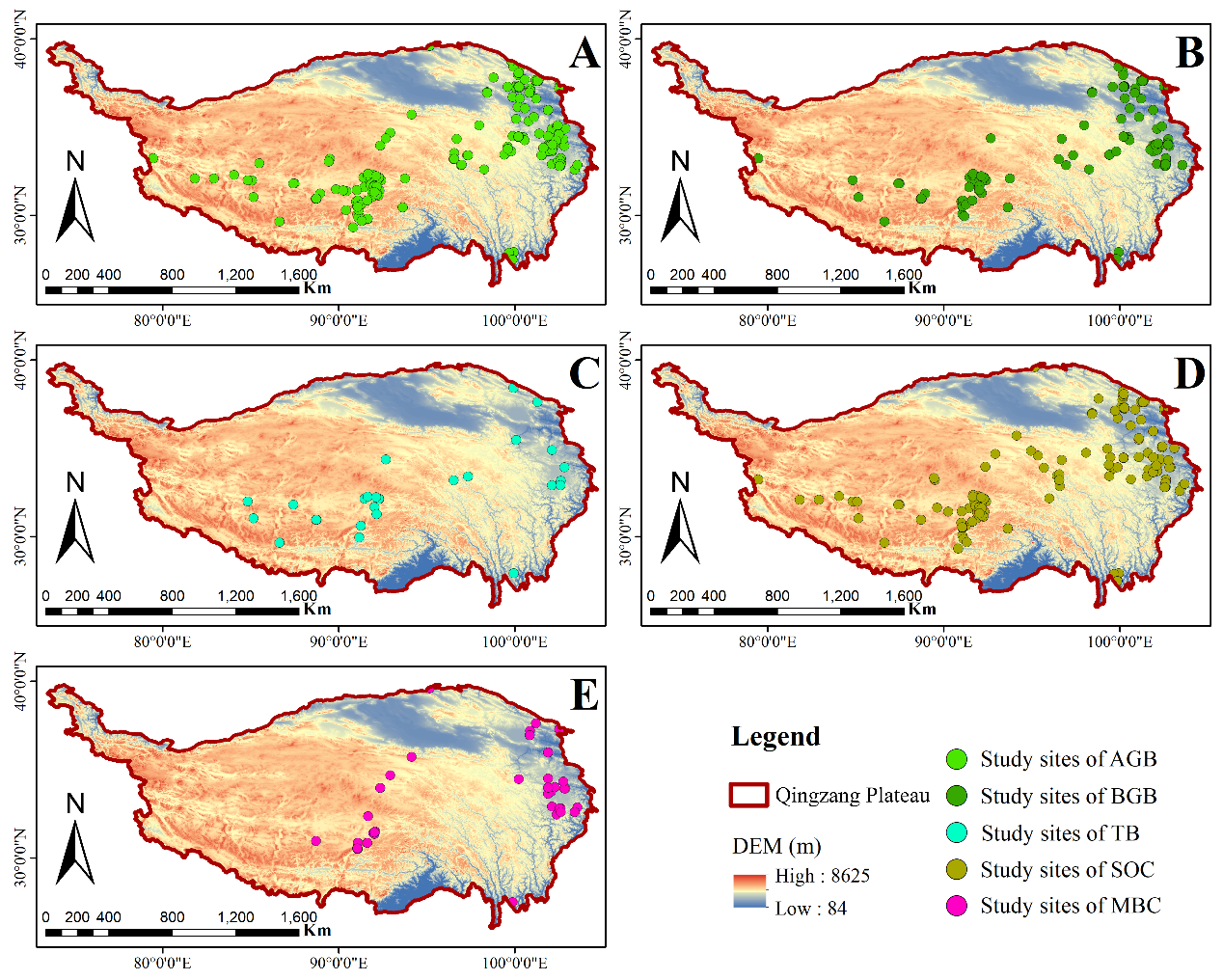


**Fig. S1** The location of study sites on the Qingzang Plateau. Graph (A), (B), (C), (D), and (E) contains aboveground biomass (AGB), belowground biomass (BGB), total biomass (TB), soil organic carbon (SOC), and microbial biomass carbon (MBC), respectively.
